# Rapid Innovation of Precision Psychiatry Interventions Using Dynamical Systems Modeling and Ecological Quasi-Experiments: Protocol for a Proof-of-Concept Study on Eating Disorders

**DOI:** 10.2196/104849

**Published:** 2026-10-15

**Authors:** Emily K Presseller, Falzah Farooq, Estefano Reyes-Madriz, Marianne Rouleau-Tang, Nicholas C Jacobson, J Graham Thomas, Lisa A Marsch

**Affiliations:** 1 Center for Technology and Behavioral Health, Geisel School of Medicine, Dartmouth College, 46 Centerra Pkwy, Suite 300/301, Lebanon, NH, 03766, United States, 1-603-646-7109; 2 Department of Biomedical Data Science, Geisel School of Medicine, Dartmouth College, Hanover, NH, United States; 3 Department of Psychiatry, Geisel School of Medicine, Dartmouth College, Hanover, NH, United States; 4 Department of Psychiatry and Human Behavior, Alpert School of Medicine, Brown University, Providence, RI, United States; 5 Weight Control and Diabetes Research Center, Miriam Hospital, Providence, RI, United States

**Keywords:** digital intervention, ecological momentary assessment, sensor, eating disorder, anorexia nervosa, bulimia nervosa, binge eating disorder, mHealth, precision psychiatry, personalization, mobile phone, mobile health

## Abstract

**Background:**

Nearly 83 million Americans experience mental illness, but fewer than 50% accessed past-year mental health services. Disseminable, potent mental health interventions are critically needed. Standalone digital interventions offer promise for improving clinical outcomes, but the efficacy of these interventions to date is modest, likely due in part to insufficient personalization to patient heterogeneity, varying context, and the stochastic nature of mental health symptoms.

**Objective:**

This study seeks to improve the efficacy of precision digital health interventions by developing real-world, person-specific models for psychopathology using ecological data and dynamical systems modeling. Dynamical systems modeling of ecological momentary assessment (EMA) and smartphone sensor data will provide insight into each individual’s maintenance system for psychopathology in their daily life. Just-in-time adaptive interventions (JITAIs) provide support in the moment at specific decision points, often via smartphone, when the risk for maladaptive behaviors is determined to be elevated. This study will use microrandomized assignment to deliver JITAIs at times of risk for maladaptive behavior based on the individual’s dynamical system to evaluate the granular effects and mechanisms of specific therapeutic skills. Proof-of-concept of these methods for improving potency of precision psychiatry digital interventions will be evaluated in eating disorders (EDs), as EDs are characterized by easily measurable maladaptive behaviors, maintained by a complex intersection of biopsychosocial factors, and their frontline treatment, enhanced cognitive behavioral therapy (CBT-E), is primarily behavioral. The aims are to (1) test the hypothesis that person-centered dynamical models will describe risk for dietary restriction, binge eating, and compensatory behaviors with ≥60% average area under the curve; (2) test hypothesized effects and mechanisms of CBT-E skills using JITAIs; and (3) characterize feasibility and acceptability of the standalone JITAI system.

**Methods:**

The study will enroll 170 adults with EDs. EMA and smartphone sensor data measuring CBT-E risk factors will be collected for 16 weeks. Participants will receive JITAIs delivered based on their idiographic risk models for 10 weeks. ED symptoms, feasibility, and acceptability data will be collected at the end of the study. The study was registered on ClinicalTrials.gov on May 19, 2026. The first participant was enrolled on July 8, 2026.

**Results:**

This project is funded from August 2025 to July 2030. Recruitment will occur from June 29, 2026, to July 2029. Data collection started in July 2026 and will end in November 2029. A total of 4 participants have been enrolled (as of August 14, 2026). Results will demonstrate the efficacy of idiographic dynamical systems modeling for accurately describing real-time behavioral risk and provide insight into the relative effects and mechanisms of key CBT-E skills under conditions of daily life.

**Conclusions:**

The study will set the stage for a future randomized controlled trial to evaluate the optimized, standalone JITAI, which will have the potential to substantially improve access to evidence-based mental health care.

## Introduction

### Background

The United States is experiencing a mental health crisis. Approximately 33% of US adults (nearly 83 million individuals) live with a mental health disorder [1], which is linked to increased rates of all-cause mortality [2], suicide [2], substance use [1], physical health problems [3], and impaired quality of life [4]. Fewer than 50% of Americans with mental health disorders accessed any past-year treatment for these disorders [1]. Limited use of mental health services may be due in part to insufficient health care resources (ie, services, providers, and settings) to meet mental health care needs [5] and barriers to accessing treatment in traditional health care settings (eg, lack of health insurance and stigma) [1]. There is an urgent need for low-burden, highly accessible, and potent mental health interventions.

Precision psychiatry digital interventions offer significant promise for improving mental health outcomes but require optimization to realize their promise. Precision psychiatry, which uses data to personalize treatments based on patient heterogeneity, offers substantial promise for improving mental health outcomes and reducing the associated public health burden when delivered at scale [6]. Precision psychiatry efforts using data to inform decision-making about treatment modality, approach, and intensity have yielded significant improvements in clinical outcomes for a range of mental health disorders compared with treatment-as-usual [7]. However, effect sizes associated with treatment personalization remain small [7], likely due in part to (1) insufficient personalization to the range of factors that impact clinical presentation and treatment response, (2) personalization using group-level decision rules that fail to account for idiographic (ie, person-specific) differences in disorder maintenance, (3) reliance on static interventions that do not address within-person variability in clinical needs over time, and (4) insufficient precision of delivered intervention content due to limited understanding of the effects of individual treatment components on outcomes and maintaining factors (and how these effects vary across individuals). To move precision treatments forward and achieve their full promise, the psychiatry field must address these limitations, which this study proposes to do.

First, the potency of precision interventions may be increased by comprehensive systems modeling of clinical maintenance factors. Comprehensive assessment of the factors that maintain psychopathology is essential to personalize psychiatric interventions to the individuals who receive them. Maintenance of psychopathology over time can be described by a system of interrelated factors that impact each other and are governed by system processes including inputs and outputs of maintenance variables, feedback loops, inertia, and resiliency [8,9]. For example, many individuals experience variable levels of body image concerns (in frequency and intensity) in day-to-day life. An individual predisposed to an eating disorder (ED) may experience momentarily increased body image concerns (eg, due to comparing their body unfavorably to another’s at the beach), which leads them to engage in dietary restriction (eg, calorie counting). This restriction leads to weight loss in the following days, which provides a sense of accomplishment and reduces their body-related distress, thereby reinforcing the dietary restriction. In turn, this feedback loop increases the frequency and intensity of dietary restriction, yielding more weight loss, which continues to reinforce the cycle. To date, most precision psychiatry efforts have personalized interventions based on only 1 between-subjects variable (eg, symptom severity and presence or absence of comorbidity), failing to address the potential interactions between variables [7]. The Marburg Declaration, a collection of recommendations by experts in clinical psychology for improving mental health interventions, identified systems modeling of multiple related mechanisms as a primary target for improving psychological treatment efficacy [10]. Precision psychiatry efforts that personalize interventions to the complex system of contributing factors and their interactions could improve intervention potency by targeting intervariable system dynamics. Despite this promise, no previous studies have harnessed the power of comprehensive, dynamical systems modeling to understand real-time, real-world maintenance of mental illness.

Furthermore, person-specific modeling of maintenance systems is likely to improve treatment efficacy compared with between-subjects modeling. Comprehensive assessment and systemic group-level modeling of maintenance factors for psychopathology alone are insufficient to optimize precision psychiatry efforts. Rather, the field must also account for individual differences in maintenance systems for psychopathology and personalize interventions based on idiographic (person-specific) maintenance systems. Research using idiographic models to understand mental health-related experiences such as stress [11], emotions [12,13], depression [14,15], social anxiety [16], and substance use craving [17-19] has identified substantial between-person variability in the direction and magnitude of relationships between established group-level maintenance factors and outcomes, suggesting that group-level models fail to accurately characterize these associations for many individuals. Precision interventions that target idiographic maintenance of psychopathology have high potential to improve clinical outcomes, cost-effectiveness, and efficiency [20-23]. However, interventions informed by idiographic maintenance models have not been widely developed, likely due to the need for repeated assessment over time to reliably estimate idiographic models [24]. Ambulatory data collection (eg, ecological momentary assessment [EMA] and passive sensing) in which variables are assessed as often as multiple times per day for weeks or months could provide the intensive time series data on maintenance factors and symptoms needed for idiographic modeling [24]. Accounting for individual differences in relevant symptoms, maintenance factors, and their associations by using idiographic modeling to tailor precision psychiatry interventions is likely to improve the efficacy of these interventions by targeting the specific mechanisms most relevant to the patient.

Adaptive interventions, tailored to an individual’s momentary contextual circumstances, may increase efficacy and maintain participant engagement with precision psychiatry tools. Although personalization of psychiatric interventions based on comprehensive, idiographic, dynamical systems models of symptom maintenance is likely to improve upon extant personalization efforts, mental illnesses are often influenced by factors that vary day to day and can change drastically over time. For instance, dietary restriction (eg, skipping meals) increases risk for binge eating later that day via increases in hunger signaling. The impact of dietary restriction on risk for binge eating is likely magnified when a patient is weight suppressed (ie, weighing less than their lifetime highest adult weight) due to the neurobiological signals that attempt to restore weight homeostasis. Therefore, while only prolonged dietary restriction (eg, >8 h without eating) may increase risk for binge eating when a patient is at their highest weight, if a patient with an ED loses weight over time due to chronic undereating, shorter periods of dietary restriction (eg, >4 h without eating) may increase binge eating risk and warrant in-the-moment intervention. An adaptive intervention system should thus be able to (1) assess and intervene on in-the-moment maintenance factors (eg, dietary restriction) and (2) change the criteria for intervening on these factors as other maintaining factors (eg, weight) change over time. Precision interventions that integrate comprehensive, real-time, repeated assessment of maintaining factors for psychopathology are likely to demonstrate magnified effects due to granular tailoring of interventions to a patient’s specific momentary circumstances and to longitudinal changes in the system of factors maintaining psychopathology.

Just-in-time adaptive interventions (JITAIs), which are interventions adapted based on data and delivered at critical times of risk for maladaptive behaviors or symptoms, constitute an important first step toward tailoring treatments to momentary circumstances [25]. These interventions, which have been applied to depression [26,27], anxiety [27], EDs [28,29], and substance use disorders [30], recognize that individuals’ level of symptoms and risk change over time (often on the scale of minutes to hours) and typically capture this variability using repeated, ecological data collection methods such as EMA and sensors. However, extant JITAI systems largely use group-level decision rules to deliver interventions, likely yielding interventions that sometimes feel irrelevant or inappropriate to individual patients. Existing JITAI systems also use often fixed decision rules (ie, rules or algorithms that are determined at the onset of treatment and then applied to momentary data) which do not account for longitudinal shifts in the system that may occur, potentially undermining intervention efficacy and patient engagement. Systems modeling combines the strengths of data-driven analyses like machine learning in terms of accuracy, while yielding more interpretable models that account for processes maintaining (or conversely, disrupting) system homeostasis, such as feedback loops. Developing JITAI systems that are based on idiographic maintenance models and adapted to day-to-day variability in maintenance factors and longitudinal changes in the system dynamics could promote user engagement by increasing perceived utility of the interventions while minimizing patient burden.

Finally, precision psychiatry efforts would be improved by granular understanding of the effects and mechanisms of individual treatment components that comprise evidence-based treatment packages. In addition to developing idiographic precision interventions that adapt to varying patient needs, identifying which interventions to deliver when will further optimize precision psychiatry interventions. Psychotherapeutic interventions have historically been evaluated as packaged treatments delivered over weeks to months and their effects have been measured infrequently (eg, mid-treatment, post treatment, and follow up). Recently, researchers have begun to conduct dismantling trials to evaluate the individual and synergistic effects of specific treatment components, for instance via full factorial study designs [31]. However, these studies still evaluate treatment effects aggregated over weeks or months (from pre- to posttreatment), despite effects likely occurring on much shorter timescales during treatment (ie, after using a therapy skill, patients likely experience near-immediate changes to the variables relevant to the system maintaining their symptoms). For instance, when a patient with binge eating uses urge surfing to tolerate (rather than act on) their urges to binge, they likely experience an immediate increase in self-efficacy and perceived control over their eating and avoid the increased weight concerns that typically follow binge episodes. These impacts may reduce dietary restriction the next day (due to lack of perceived need to compensate for binge eating), which in turn decreases the patient’s future binge eating risk. Avoiding the binge episode constitutes a direct improvement in ED symptoms, while improved self-efficacy and decreased dietary restriction reduce future risk. Cognitive behavioral therapy (CBT), the leading evidence-based psychotherapeutic treatment for a breadth of mental health disorders including depression [32], anxiety disorders [33], obsessive-compulsive and related disorders [34], trauma-related disorders [35], substance use [36], and EDs [37], supports patients in changing their behaviors and thinking patterns to improve psychological symptoms. CBT is skills-based and amenable to delivery in-the-moment when patients may particularly benefit from using certain behavioral or cognitive skills. In this way, JITAIs could be conceptualized as *quasi-experimental manipulations* of patient behavior to facilitate testing the short-term impacts of individual treatment skills. These prompts may be delivered to induce treatment skill use at each decision point (ie, at each point when the criteria for JITAI delivery are met), the effects on symptoms and maintenance factors of which could then be measured in real time using repeated ecological data collection. Microrandomizing the delivery of JITAIs involves randomizing the content of JITAI delivered at the decision point within participants, such that all participants have the opportunity to receive all types of JITAIs. Microrandomization of JITAIs will allow for evaluation and comparison of the immediate effects of these treatment skills on symptoms of interest to understand the most potent treatment ingredients under real-world conditions. Despite the promise of this use of microrandomization of JITAIs for improving understanding of CBT efficacy, only a few previous studies have evaluated the immediate effects of individual CBT skills on maintaining factors for mental illness in patients’ daily lives, and none of these studies have used microrandomization to do so experimentally [38,39]. These studies found evidence of differential effects of specific CBT skill use that followed from ecological momentary interventions on proximal clinical improvements [38,39], indicating that using JITAIs to quasi-experimentally test the effects and mechanisms of CBT skills may improve intervention precision.

Standalone digital intervention formats permit precision psychiatry interventions to be delivered at scale. Standalone smartphone interventions can be implemented broadly as they are low cost, accessible to anyone with a smartphone (ie, 90% of Americans) [40], and do not require access to mental health providers. These benefits could overcome primary reported barriers to accessing mental health care, including cost, limited mental health resources, geographic proximity, and social stigma [5]. As these barriers disproportionately impact low- and mid-income individuals and individuals living in rural areas [5], increasing access to effective mental health care is a critical step toward addressing health disparities. Optimization of standalone digital interventions via tailoring of intervention targets to idiographic systems of symptom maintenance, intervention timing to ecological circumstances of risk, and intervention content using empirical evaluation of the effects of individual treatment skills is likely to enhance the clinical effects of these low-intensity interventions, which (at scale) could yield large improvements in mental health outcomes [6]. This study will lay the groundwork for future evaluation of an optimized, standalone precision psychiatry smartphone intervention.

EDs are an ideal population in which to test proof-of-concept of using ecological data collection, idiographic dynamical systems modeling, and micro-randomized JITAIs to efficiently produce an optimized, precision psychiatry digital intervention. EDs, including anorexia nervosa, bulimia nervosa, and binge-eating disorder, are complex psychiatric illnesses characterized by disturbances in eating behaviors (eg, restriction of dietary intake and binge eating), maladaptive weight control behaviors (eg, self-induced vomiting and laxative misuse), body image disturbances, and cognitive symptoms (eg, preoccupation with eating and fears of weight gain from specific foods) [41]. EDs are associated with substantial physical and psychological morbidity, including premature death [42,43], cardiovascular problems [44,45], type 2 diabetes [46], and comorbid psychiatric disorders [46,47]. Previous literature substantiates that EDs are influenced and maintained by numerous biological (eg, appetite regulation processes), psychological (eg, body image concerns and emotion regulation), and social factors (eg, interpersonal conflict) [48-50]. Further complicating the clinical picture, many of these factors demonstrate notable momentary variability (ie, they fluctuate regularly), are interrelated (such that fluctuations in one impact others), and change over time as the ED progresses and becomes entrenched [51]. The frontline treatment for EDs in adults, enhanced cognitive behavioral therapy (CBT-E), uses a transdiagnostic model [52]. The CBT-E model conceptualizes ED maintenance via 2 cycles; in the first, body image concerns (influenced by low self-esteem and clinical perfectionism) produce attempted or actual dietary restriction and other inappropriate weight control behaviors, which increase vulnerability to binge eating to maintain nutritional and weight homeostasis; in the second, aversive momentary mood (which may result from interpersonal problems or other life stressors) increases risk for binge eating, which temporarily improves mood but subsequently increases risk for attempted or actual dietary restriction and compensatory behaviors to reduce the impact of binge eating on shape or weight (and associated guilt). CBT-E, like other CBT treatments, is skills-based and targets patients’ eating behaviors, thinking patterns, and responses to mood states to reduce ED symptoms [52]. EDs are well-suited for evaluating the benefits of idiographic modeling, ecological tailoring, and experimental testing of individual intervention components to advance precision psychiatry given the clearly measurable nature of primary ED behaviors (ie, dietary restriction, binge eating, and compensatory behaviors), complexity of biopsychosocial factors contributing to their maintenance, and skills-based nature of the frontline treatment. An estimated 80% of individuals with EDs have not received treatment [53] due to financial barriers, lack of access to specialized clinicians, geographic barriers, and stigma [54], indicating that this population is also likely to be interested in and benefit from a standalone digital intervention.

### This Study

The study will enroll 170 adults with EDs who will complete EMA and smartphone sensor data collection and receive JITAIs to use treatment skills at times of elevated risk for ED behaviors. The specific aims are as follows: the first aim is to develop dynamical models describing risk for dietary restriction, binge eating, and compensatory behaviors using ecological data and dynamical systems modeling. We hypothesize that these models will demonstrate adequate (ie, ≥ 60%) average area under the curve. The second aim is to use microrandomized JITAIs to understand the relative effects and mechanisms of CBT-E skills on ED behaviors and primary ED maintenance factors (ie, body image concerns, cognitive dietary restraint, and momentary mood) under varying real-life circumstances. We hypothesize that reducing dietary restraint skills will demonstrate the largest effect on dietary restriction and urge management skills will demonstrate the largest effect on binge eating and compensatory behaviors. Figure 1 depicts hypothesized mediation pathways by which CBT-E skills are expected to impact ED behaviors. Finally, the third aim is to characterize preliminary feasibility and acceptability of the delivered JITAIs. We hypothesize that the JITAIs will meet a priori feasibility and acceptability standards.

**Figure 1.**
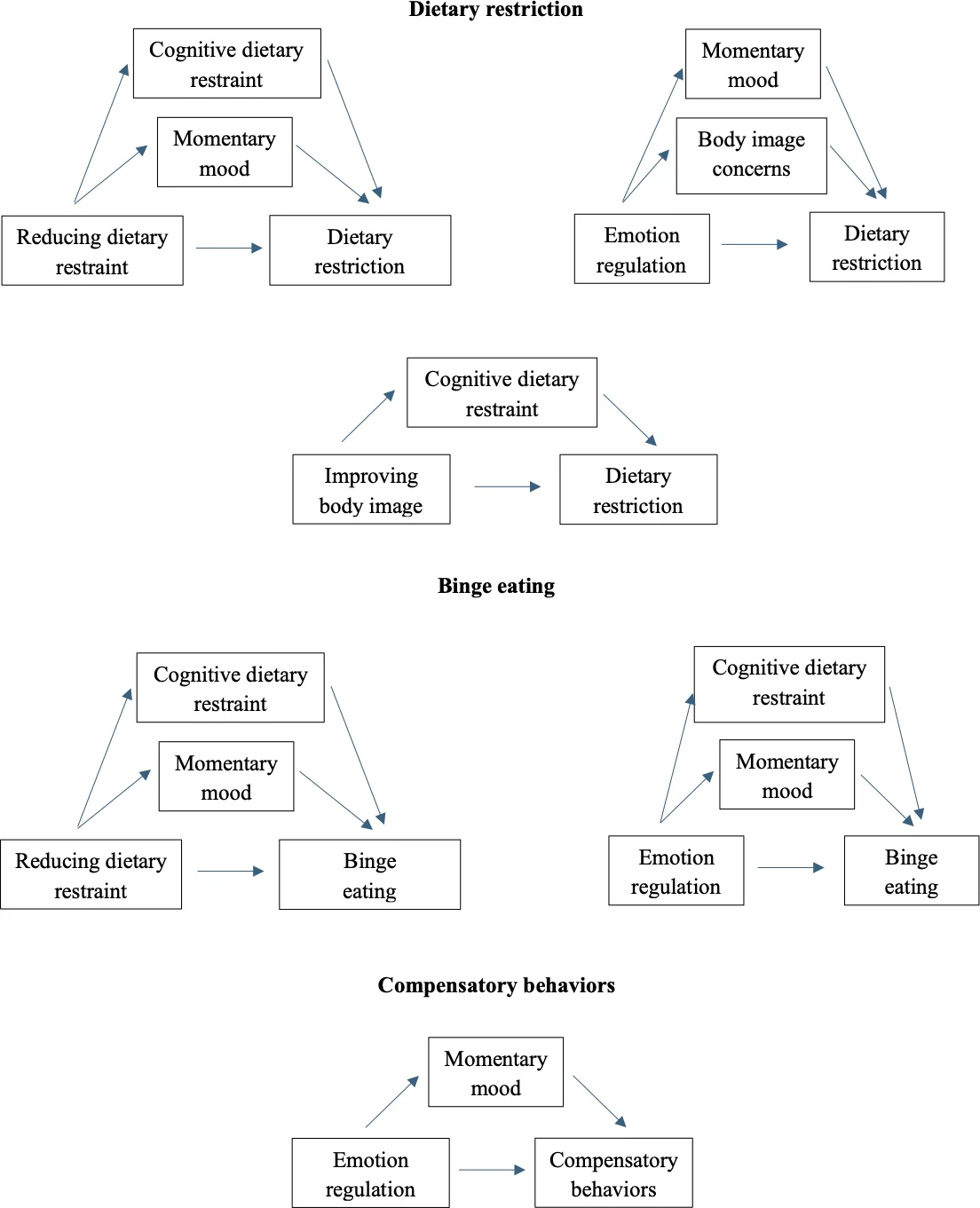
Hypothesized mediation pathways explaining the effects of primary enhanced cognitive-behavioral therapy skills on eating disorder behaviors.

## Methods

### Participants

Participants will be 170 adults with clinically significant EDs. Inclusion criteria are (1) age 18‐70 years, (2) recurrent engagement (≥12 instances) in at least 1 type of ED behavior (ie, binge eating, dietary restriction, or compensatory behaviors) in the past 3 months, (3) ownership of a smartphone, and (4) willingness to complete EMA and sensor data collection. Exclusion criteria are (1) inability to fluently speak, read, and write in English, (2) BMI <17.5 kg/m^2^, (3) medical complications of ED symptoms requiring immediate treatment, (4) current ED-focused psychotherapy, and (5) intellectual disability or severe psychopathology inhibiting engagement in study protocols. Informed consent will be obtained from all participants. As EDs are approximately twice as common among females as males [55], we anticipate recruiting females and males in a 2:1 ratio. It has been well-established that EDs impact individuals across all racial and ethnic groups [56]. However, existing research in EDs has included predominantly non-Hispanic White individuals, limiting generalizability of findings and unduly excluding individuals of racial and ethnic minority identity from receiving potential research benefits. To counteract this historical legacy, we will implement targeted recruitment to intentionally include a significant number of individuals belonging to racial and ethnic minority groups. The study will aim to recruit a sample comprised of 85 (50%) non-Hispanic White participants and 85 (50%) participants belonging to racial and/or ethnic minority groups.

### Recruitment

Participants will be recruited nationally across the United States using social media advertising, other online advertising, and ResearchMatch (Vanderbilt University Medical Center). Participants will complete a phone screen followed by a virtual baseline assessment (conducted via HIPAA [Health Insurance Portability and Accountability Act]-compliant Microsoft Teams call) to confirm eligibility and be oriented to data collection procedures. The study was registered on ClinicalTrials.gov (NCT07600671) as of May 19, 2026, and is currently recruiting. The first participant was enrolled on July 8, 2026.

### Data Collection Procedures

Following determination of eligibility, participants will then complete a 16-week data collection procedure. Participants will complete EMA and collect smartphone sensor data throughout the 16-week data collection period; the first 4 weeks of data collected will be used to develop idiographic dynamical systems models to describe the participants’ risk for ED behaviors of relevance. The performance of these models will be evaluated using weeks 5 and 6 of the participants’ ecological data. Participants will receive JITAIs, delivered based on their own idiographic dynamical systems models, for the remaining 10 weeks. Participants will complete an end-of-study visit to evaluate feasibility and acceptability of the JITAIs and collect preliminary efficacy data on the intervention package.

### Measures

#### Eating Disorder Examination

The Eating Disorder Examination (EDE), a valid and reliable interview for the assessment of ED symptoms [57], will be conducted at the baseline assessment to confirm eligibility and measure baseline ED pathology. ED behaviors that will count toward eligibility include objective and subjective binge eating episodes, compensatory behaviors (ie, self-induced vomiting, laxative misuse, diuretic misuse, excessive exercise, chewing and spitting, and other extreme weight control behaviors), and instances of dietary restriction (ie, skipped meals, avoidance of specific foods, limiting overall amount eaten at meals or snacks, and 8-hour fasting); participants will have to report at least 12 instances of ED behaviors falling into a singular category for eligibility to ensure adequate power for the development of idiographic models. The EDE assesses the presence of cognitive (ie, intended, successful, or not) dietary restraint, so questions will be added to assess the frequency of actual dietary restriction behaviors over the past 12 weeks. The EDE will also be administered at the end-of-study assessment to measure eating pathology following receipt of the intervention. At baseline and end-of-study, the EDE will be administered by trained research assistants who will have received didactic training, completed mock assessments, shadowed the principal investigator completing the EDE with a participant, and completed several EDEs while shadowed by trained assessors (eg, the principal investigator). To ensure adequate training and interrater reliability, new assessors will be shadowed on the EDE until they consistently rate participant responses in agreement with previously trained assessors. A subset (n=34, 10%) of participant EDEs will be randomly selected and recoded to establish interrater reliability during the study.

#### EMA

Participants will complete signal-contingent EMA surveys during waking hours to assess eating episodes, ED behaviors, cognitive dietary restraint, body image concerns, momentary mood, perfectionism, interpersonal problems, and self-esteem. These maintenance factors were selected based on their implication in the CBT-E theory of EDs [52] and their substantiated relevance to ED symptoms in previous EMA and other prospective studies [58-66]. EMA surveys will be delivered at 6 semirandom times per day via the Eating Disorder Dynamic Intervention (EDDI) app (the smartphone app developed for this study). EMA questions will be drawn from established measures (Short Form Eating Disorder Examination-Questionnaire [67], Short Form Positive and Negative Affect Schedule [68], Frost Multidimensional Perfectionism Scale [69], Social Adjustment Scale–Self-Report [70], and Single-Item Self-Esteem Scale [71]) or items previously used in EMA research in EDs and modified to assess these factors in the time since the last EMA survey [72]. Behaviors (eating episodes and ED behaviors) will be reported since the last survey on each EMA. ED maintenance factors (cognitive dietary restraint, body image concerns, momentary mood, perfectionism, and self-esteem) will be reported on 5-point Likert-type scales; interpersonal problems are reported on the specific 5-point scales retained from the Social Adjustment Scale–Self-Report. After each delivered JITAI, the following EMA survey will begin with the question, “*Since you received the suggested personalized treatment skill, have you used it?*” to confirm experimental manipulation of participant behavior by JITAIs (ie, induction of CBT-E skill use).

#### Smartphone Sensor Data Collection

To augment EMA assessment of critical maintenance factors for ED symptoms without adding substantial participant burden, sensor data will be passively collected by participants’ smartphones to measure factors associated with momentary mood (ie, accelerometry, keyboard dynamics, and location) and physical activity (accelerometry) [73-75]. Data will be collected using native smartphone sensors and accessed by the smartphone app developed for the study. Factors to be measured by smartphone sensors were selected based on relevance to the CBT-E model [52] and in previous prospective literature [58-66] and established capacity for measurement by smartphone sensors in other populations [73-75].

#### Feasibility and Acceptability

Feasibility of the JITAIs will be characterized by % of eligible participants who enroll in the study, % attrition, and % of JITAIs that yield CBT-E skills use. Acceptability questions will be derived from the technology acceptance model [76,77] and a modified version of the Perceptions of Computerized Therapy Questionnaire [78]. Acceptability ratings will be made on 7-point Likert scales (1=strongly disagree to 7=strongly agree).

### Intervention

Participants will view video modules (total 4) explaining each CBT-E skill after completing their first 4 weeks of data collection. These modules will be developed based on CBT-E guided self-help and will include rationale, didactic instruction, and interactive activities (completed and saved in the EDDI app) to prompt participants to apply the CBT-E skill content to their own symptoms and lives. Participants will have constant access to psychoeducation about CBT-E skills within the EDDI app and this psychoeducation will be hyperlinked directly in all JITAIs. Starting in week 7 of data collection, JITAIs designed to prompt in-the-moment use of CBT-E skills (reducing dietary restraint, urge management, emotion regulation, and improving body image; refer to Table 1 in Multimedia Appendix 1) will be delivered via smartphone push notification when participants are at elevated (≥50%) risk for dietary restriction, binge eating, and/or compensatory behaviors based on their own personalized risk models. Although the threshold of 50% risk is higher than has been used in previous JITAI studies, it was selected to maximize the magnitude of observed clinical effects of therapeutic skills and minimize fatigue effects associated with JITAIs delivered too often [25]. At each identified instance of risk, individual participants will be microrandomized to receive 1 of the 4 types of JITAIs (Table 1 in Multimedia Appendix 1) or no JITAI (as a control condition) with equal randomization probability for each condition. This is to ensure that all participants have the opportunity to receive JITAIs targeting all CBT-E skills during the study. To avoid experimental contamination, participants will receive a maximum of 1 JITAI per day.

### Data Analysis

#### Aim 1

System identification methods [79] will be used to estimate idiographic dynamical systems models from the first month of EMA and smartphone sensor data collected from each participant separately. Distinct models will be developed to describe risk for dietary restriction, binge eating, and compensatory behaviors for each participant as relevant, as individual participants may exhibit differing combinations of these behaviors, and extant evidence suggests that maintenance factors and their interactions differ between ED behavioral outcomes [65]. Antecedents and behavioral functions are similar across types of dietary restriction (eg, skipped meals, restriction of amount consumed, and avoidance of specific foods for shape or weight reasons) [52], for objective- and subjectively-large binge eating episodes [52,80], and across types of compensatory behaviors (eg, self-induced vomiting, laxative misuse, and excessive exercise) [52,81], and individuals with EDs often engage in multiple types of dietary restriction [82,83], binge eating episodes [84], and compensatory behaviors [85]. Accordingly, these specific subtypes of ED behaviors will be aggregated into within-person categories of dietary restriction, binge eating, and compensatory behaviors to maximize data for model development.

System identification involves identification of candidate equations that could be used to model the dynamics in the outcome of interest based on inputs of interest, fitting the models, and validating the models against an a priori criterion of fit [79]. Candidate equations for modeling risk for dietary restriction, binge eating, and compensatory behaviors will be derived from CBT-E theory. Then, a nonlinear autoregressive exogenous model (NARX) will be used in combination with candidate equation structures to estimate system parameters, consistent with the “gray box” modeling approach within systems engineering [86]. NARX was selected given its flexibility for system identification incorporating both known model structures and unknown model terms and allowance of both linear and nonlinear terms [86]. Input variables will include eating episodes, cognitive dietary restraint, body image concerns, momentary mood, perfectionism, interpersonal problems, self-esteem, and average adherence to EMA and smartphone sensor data collection. A continuous time framework will be used for modeling the associations between these inputs and ED behaviors. Input variables will be selected for each participant’s model using elastic net regression with rolling origin cross-validation for stability selection given limited time points per person and autocorrelation of the data. A sensitivity analysis will compare the results of elastic net for variable selection across the first 20 participants to an approach in which input variables are sequentially added to the model in order of theoretical and empirical importance in previous literature, and inputs will be retained if they significantly improve model fit (ie, minimize Akaike Information Criterion) while reducing model complexity, evaluated using a Pareto analysis [87]. If the sensitivity analysis exhibits significant divergence between elastic net and sequential variable selection, model performance will be compared, and the modeling approach will be adjusted subsequently.

Model development and initial testing (ie, to determine model structure, estimate model parameters, and decide which input variables to retain) will be conducted using data collected during the first 4 weeks of the data analysis period; model parameters will be tuned using rolling-origin cross-validation, with an estimated 118 observations per participant in the training dataset, given 6 EMA surveys/day × 7 days/week × 4 weeks × 0.7 compliance . We conservatively anticipate approximately 10% of the EMA surveys (ie, approximately 3 surveys/week, given 6 surveys/day × 7 days/week × 0.7 compliance) will include reported engagement in each of the ED behaviors relevant to the individual, based on reported behavioral frequencies during other EMA studies of individuals with EDs conducted by the study team in which average binge eating frequencies ranged from 4.1 to 6.5 per week, average compensatory behavior frequencies ranged from 2.2 to 10.2 per week, and average dietary restriction frequencies ranged from 1.2 to 7.3 per week. The developed models will be validated against the benchmark of 60% area under the receiver operating characteristic curve (AUROC) for their accuracy in describing the ED behavior using the data collected during the first 2 weeks of month 2 (estimated to be approximately 59 data points per participant given 6 EMA signals/day × 7 days/week × 2 weeks × 0.7 compliance); this will ensure that AUROC is evaluated on previously unseen data to avoid inflated fit metrics from overfitting. Given the comprehensiveness of maintenance factors assessed and demonstrated accuracy of dynamical models developed to describe other health phenomena [88,89], we anticipate our models’ average AUROC will surpass 60%. To characterize model performance for implementation of JITAIs, the frequency of ED behaviors occurring when the risk was predicted to be low (ie, <50%) will be calculated across the 10 weeks of JITAI delivery by participant.

Because aim 1 of the study aims to identify models that describe the maintenance of ED behaviors (ie, it is a matter of system identification), the traditional concept of power to test the probability of certain effects and compare this probability to an established significance level (ie, hypothesis testing) is not relevant. Instead, this aim can be thought of as a series of idiographic (n=1) studies from which we can seek to draw more broadly generalizable conclusions. Given the idiographic nature of this approach, increasing power (ie, data) can be accomplished by increasing frequency of sampling and duration of data collection within each individual participant. A total of 6 EMA signals per day (which we expect to produce at least 4 completed EMA surveys per day, based on conservatively estimating approximately 70% EMA compliance) is expected to produce sufficient data to generate meaningful dynamical models for each individual participant based on previous applications of these modeling procedures by members of the study team.

#### Aim 2

The effects of CBT-E skills use that results from delivered JITAIs will be evaluated using multilevel models using all aggregated participant data to maximize power. Multilevel logistic regression models, in which the CBT-E skill condition (ie, reducing dietary restraint and restriction, urge management, emotion regulation, improving body image, or no CBT-E skill) is the independent variable and ED behaviors (dietary restriction, binge eating, and compensatory behaviors) are the outcomes, will be tested. Only instances when participants reported implementing CBT-E skills in response to delivered microrandomized JITAIs will be included in aim 2 analyses. ED behavioral outcomes will be measured at the EMA time point following the delivery of each JITAI that successfully induces CBT-E treatment skill use. Missing data will be addressed with single imputation. Observations will be nested within participants to account for between-person variability in the effects of CBT-E skills, and random intercepts and slopes will be included in the model unless doing so inhibits model convergence. Random intercepts and slopes will be extracted to characterize between-person variability in effects of CBT-E skills on ED behaviors. *P* values<.05 will be considered statistically significant. Odds ratios will be calculated as a measure of effect size, and medium effects (odds ratios≥1.5) will be considered clinically meaningful.

To evaluate mediators of the effects of CBT-E skills on ED behaviors, multilevel mediation models will be conducted. In each mediation model, the independent variable will be the CBT-E skill of interest (or control observations where no CBT-E skill was prompted), the mediator will be 1 of the 3 mediators of interest (body image concerns, cognitive dietary restraint, and momentary mood), and the dependent variable will be 1 of the 3 ED behaviors (ie, dietary restriction, binge eating, or compensatory behaviors); 36 total mediation models will be conducted. In each case, CBT-E skill use will be measured at the EMA survey after JITAI delivery (as described in the previous sections), the mediator will be measured at the same EMA time point, and the ED behavior will be measured at the following EMA time point (ie, 1 time point after the JITAI induced CBT-E skill use). Observations will again be nested within participants with random slopes and intercepts. Random slopes and intercepts will be extracted from mediation models to characterize between-person variability in mechanisms of CBT-E skills. The Holm-Bonferroni method will be used to adjust *P* values indicative of statistical significance to control the family-wise type I error rate given the number of models examined while minimizing the increase in type II error [90].

#### Aim 2 Power

To determine the need to test the effects of CBT-E skills use on ED behaviors with power=0.80, the sample size for microrandomized trial applet was used [91]. When conducting the power analysis, we inputted a study duration of 70 days (based on the 10-week period when participants will receive microrandomized JITAIs) and estimated 4 decision points per day (conservatively expecting 4 completed EMA surveys per day, assuming approximately 70% compliance × 6 survey signals/day). Randomization probability was constant at 0.20 (indicating the rate of randomization to any of the 5 JITAI conditions based on equal randomization probability). We estimated that availability for microrandomization would linearly decrease over the course of the study, due to improvements in ED behavior risk (as patients benefit from using CBT-E skills). We estimated the initial expected availability for microrandomization to be 0.10 (indicating that participants would be identified to be at >50% risk for an ED behavior at approximately 10% of surveys at the start of the study, assuming 3 ED behaviors/week (as described in the previous sections) and 28 total completed surveys/week (6 surveys/day × 7 days/week × 0.7 compliance) and the average expected availability to be 0.05 (indicating that participants would be identified at >50% risk for a disordered eating behavior at approximately 5% of surveys across the entire study period). These estimates are based on the reported behavioral frequencies during other EMA studies of individuals with EDs conducted by the study team. Finally, we estimated that the proximal treatment effect would be linearly increasing across the study (due to practice effects increasing the efficacy of CBT-E skill use), with an initial effect size of 0.10 and an average effect size of 0.20. The effect size estimate for the relationship between CBT-E skills and ED behaviors (0.20) was based on the limited previous research examining the association between CBT-E skill use and ED behaviors over a full course of treatment, which identified effect sizes between 0.19 and 0.34 [92]. This yielded a required sample size of 148 participants to be powered at 0.80 to detect significant effect (*P*<.05). Estimated EMA compliance is based on previous research using EMA among individuals with EDs, which has identified average missing data rates of 4.4%‐32.6% [58,59,61,63-66,93-95].

To determine the sample size needed to detect mediation effects with power=0.80, a power analysis was conducted using MedPower [96]. A small (ie, 0.20) effect was assumed between independent (CBT-E skills) and dependent variables (ED behaviors), and a slightly smaller (ie, 0.15) effect was assumed both between the independent variable and mediator and between the mediator and dependent variable. As above, the effect size estimate for the relationship between CBT-E skills and ED behaviors (0.20) was based on the limited previous research examining the association between CBT-E skill use and ED behaviors over a full course of treatment [92]. This indicated that 446 observations were needed to have 0.80 power to detect a mediation effect. Assuming that participants will receive microrandomized JITAIs at 5% of completed surveys (6 surveys/day × 0.7 compliance×70 days=294 surveys) across the 10-week period (ie, each participant will receive approximately 15 microrandomized JITAIs) and that JITAI content will be evenly distributed across the 4 CBT-E skills and the control condition, participants are estimated to receive an average of 3 instances of each type of JITAI. Given that power in EMA studies depends on the total number of observations (rather than the number of subjects), we then divided 446 by 3 instances per participant of each type of JITAI to determine the total required sample size, which yielded 149. To account for possible attrition, we increased the largest sample size yielded by the conducted power analyses (n=148‐149) by 15% to have a final sample size of 170 participants.

#### Aim 3

Descriptive statistics (percentages or means, SDs, and ranges) will be computed for all feasibility (ie, eligible participants enrolled, attrition rate, rate at which participants report implementing CBT-E skills in response to delivered JITAIs) and acceptability (ie, technology acceptance model questions, modified Perceptions of Computerized Therapy Questionnaire) metrics across the full sample of participants. Benchmarks of ≥85% of eligible participants enrolled, <15% attrition, and JITAIs inducing CBT-E skill use ≥80% of the time will be considered to demonstrate feasibility. Mean ratings ≥5 (on the 7-point Likert rating scales) on acceptability items will be considered to indicate acceptability. As additional exploratory analyses, the rate at which participants report implementing CBT-E skills in response to JITAIs will also be computed by JITAI category and by participant to inform potential future adaptation of JITAI delivery based on participant response in addition to JITAI efficacy.

### Ethical Considerations

Study procedures will be approved and overseen by the Dartmouth Institutional Review Board (IRB; initial approval received October 3, 2025, protocol number 00033481). All procedures will be conducted according to the Declaration of Helsinki.

Participants will be compensated up to US $475 for the study as follows: US $25 for the baseline assessment, US $20/week for completing data collection, with a weekly US $5 bonus for each week with EMA adherence above 80%, and US $50 for the end-of-study visit. This compensation scheme is designed to facilitate high EMA adherence despite the extended data collection period.

### Safety Monitoring

EDs are inherently associated with substantial potential morbidity and mortality, due to the impact of ED behaviors (dietary restriction, binge eating, and compensatory behaviors) and associated weight changes on physical health and to increased rates of suicidal ideation and behavior in this population. Health risks are particularly elevated among individuals with underweight (ie, BMI<18.5 kg/m^2^), individuals who have experienced recent substantial (>5%) weight loss (regardless of current weight status), individuals engaging in recurrent purging behaviors (ie, vomiting or laxative misuse), and individuals misusing medications for weight control. Several inclusion criteria and enrollment procedures will be implemented to reduce the likelihood of medical complications and suicidal behavior arising during the study. First, individuals with BMI<17.5 kg/m^2^ will be ineligible for this study, because this level of underweight requires treatment in an intensive hospital setting [97]. Similarly, participants reporting medical complications of their ED will also be ineligible for the study, as these complications warrant more intensive treatment. Participants who are at elevated risk for medical complications (ie, participants with BMI<18.5 kg/m^2^, participants who have lost >5% of their body weight in the past 6 months, participants reporting frequency of self-induced vomiting or laxative use ≥3 times per week, and participants reporting misuse of medications for weight control) will be required to visit a primary care provider for assessment of medical stability and safety to participate in a low intensity study before participation in the study. Participants who are not deemed medically stable and safe to participate by their primary care provider will not be enrolled and will be provided with local (to the participant) treatment resources. Furthermore, all participants will be required to sign a release of information to allow the study team to share information about the patient’s symptoms with their primary care provider throughout the study as needed. The release of information will be obtained before the participant’s initiation of the study daily data collection protocol. Participants endorsing active, severe suicidal ideation at the baseline assessment will be ineligible for the study; all participants endorsing any suicidal ideation at baseline will undergo a thorough risk assessment (using the Columbia-Suicide Severity Rating Scale [C-SSRS]) completed by the assessor and the principal investigator or another doctoral-level study team member, which will result in appropriate steps to ensure safety (eg, completing a safety plan, providing referrals for psychiatric or psychotherapeutic treatment in their area, instructing the participant to go to the emergency room for further assessment, and/or calling 9-1-1). All participants endorsing any suicidal ideation at baseline will be provided with the national suicide hotline information and information about accessing emergency services and will be instructed to access these services if they experience an increase in suicidal ideation during the study.

To ensure the safety of enrolled participants during their time in the study, participant EMA data will be monitored by study staff and the study safety monitor. The study safety monitor is a licensed clinical psychologist and Program Director of an inpatient specialty EDs unit who has expertise in treatment of the spectrum of adult EDs and in conducting translational research in EDs. The study safety monitor has independence in her role as safety monitor as she has no other involvement with the study. Staff will proactively contact participants who report significant changes in ED symptoms which may increase risk for medical complications. EMA data for all participants will be monitored by the research coordinator on a weekly basis to identify any increase in ED symptoms. Participants with low weight (ie, BMI<18.5 kg/m^2^ at baseline) and participants who have lost more than 5% of their body weight in the past 6 months at baseline will also be required to use a remote weight monitoring scale weekly to provide remote, blinded weights to the study team. Participants will be mailed the remote weight monitoring scale (at no cost to them) if they meet the criteria for weekly weighing during the study. If any participants exhibit a 50% or greater increase in weekly ED symptoms sustained over 4 consecutive weeks, exhibit weight loss >0.5 lbs/week for 4 consecutive weeks or >4 lbs over 4 weeks or fall below the minimum BMI of 17.5 kg/m^2^, their data will be reviewed by our independent safety monitor, who will determine if they should be withdrawn from the study. Participants who are withdrawn from the study will be provided with local (to the participant) treatment resources, and their primary care provider will be notified of their withdrawal to facilitate medical monitoring and access to adequate treatment. These safety procedures will ensure participant safety and are consistent with previous studies in anorexia nervosa and bulimia nervosa populations (eg, study by Haynos et al [98]) and with safety guidelines for outpatient treatment [97].

Adverse events that occur during the study will be reviewed and evaluated by the principal investigator within 72 hours of the study staff becoming aware of the event. Adverse events will be documented and sent to the study safety officer for review as part of the quarterly safety and recruitment reports. All serious adverse events will be evaluated by the safety officer and principal investigator within 24 hours, and all study-related serious adverse events will be reported to the IRB and National Institutes of Health within 2 weeks. All other adverse events will be reported to the National Institutes of Health and IRB on an annual basis. A provisional stopping rule related to excessive study-related adverse events will be greater than 2 per quarter.

## Results

Participant recruitment began on June 29, 2026, and will continue through July 2029, with an average anticipated rate of 5 participants per month throughout the recruitment period. As of August 14, 2026, a total of 4 participants have been enrolled, and the study is actively recruiting. All data analyses are expected to be completed by January 2030. Publication of study results is expected in July of 2030.

## Discussion

### Principal Findings

The study will yield empirically derived, comprehensive, and idiographic dynamical models of ED maintenance, extending previous work examining the individual contributions of singular variables to ED maintenance. Incorporating the breadth of maintaining factors is likely to improve model accuracy and improve the field’s understanding of the interaction effects between factors that yield ED behaviors in patients’ daily lives. The study is innovative in its application of dynamical systems modeling (an engineering approach) to understand the impact of ecological variables on the maintenance of eating pathology over time while accounting for system processes. This application of dynamical systems to ecological mental health data will constitute an important first step within the mental health field to quantify dynamics that produce the development of psychopathology, recovery, and relapse in chronic disorders. Building these models in a heterogeneous, transdiagnostic sample may also yield insight into the well-established phenomenon of diagnostic crossover (in which individuals initially diagnosed with one ED later meet criteria for other EDs).

Furthermore, the study’s application of JITAIs as quasi-experimental manipulations will facilitate a rich examination of the ecological effects and mechanisms of individual evidence-based components of CBT-E for EDs, which could transform the way the mental health field understands and implements evidence-based treatments. Despite the common understanding among psychologists that a substantial proportion of the effects of CBT are produced by patients applying therapeutic skills in their daily lives, very limited previous work has examined ecological mechanisms of therapeutic skills, and no previous study (to our knowledge) has used JITAIs to experimentally induce CBT skill use in the ecological environment. Even studies evaluating the effect of JITAI systems have almost exclusively examined their effects on a long-term (ie, after weeks or months) timescale, despite ecological data collection making proximal evaluation of these effects highly feasible. Understanding the proximal effects of individual CBT skills in daily life has the potential to both lay the groundwork for future implementation of a standalone, optimized JITAI system to extend the reach of traditional therapy and inform how clinical providers discuss treatment skill use with patients in the context of traditional therapy.

### Clinical Implications and Future Directions

The study will accrue critical data to develop and evaluate a standalone, optimized JITAI system that is highly scalable and well-suited to the complex and relapsing nature of EDs, which may improve access to treatment and clinical outcomes among underserved individuals with EDs. Principally, the study will clarify the added value of developing idiographic models for ED behavioral risk for the delivery of JITAIs. The study will also allow for the application of JITAIs that are most likely to produce improvements in behavioral risk based on an individual’s current circumstances. These data will lay the groundwork for a future randomized controlled trial evaluating the effects of the optimized standalone JITAI system for EDs relative to waitlist control.

Importantly, if effective, this intervention may be able to overcome barriers to mental health care access, which disproportionately impact marginalized and underserved groups. If proof-of-concept is established in EDs in this study, these methods could be implemented broadly in the mental health field to develop efficacious, scalable, and accessible interventions for a range of disorders (eg, depression, anxiety disorders, and substance use).

### Strengths and Limitations

The study methods are strengthened by the racial, ethnic, and gender diversity of the proposed sample, the collection of active (ie, EMA) and passive (ie, smartphone sensors) ecological data to comprehensively assess ED symptoms and their maintaining factors, the application of idiographic modeling to describe each participant’s behavior, and recruitment of a large enough sample (N=170) and extended duration of data collection to ensure that the analyses for all aims are fully powered. Study limitations include potential biases in participants who are likely interested in participating in this study (eg, preference for digital interventions, interest in and willingness to complete ecological data collection), leading to a nonrepresentative sample of individuals with EDs; importantly, similar preferences are likely to impact who uses the optimized intervention in real-world implementation as well, suggesting that the sample in this study will still likely provide meaningful information for optimization of the intervention. The study may additionally be limited by the impact of participant insight and social desirability biases on self-report of ED symptoms and their maintenance factors. The study will not enroll a control group who complete ecological data collection without receiving JITAIs; although participants will complete 6 weeks of ecological data collection before receiving any JITAIs, thereby allowing us to preliminarily understand the impact of self-monitoring on behavior, the lack of randomization precludes us from causally attributing potential improvements in ED symptoms to JITAIs specifically. Finally, the effects of JITAIs will be observed only when risk for ED behaviors is predicted to be high and may therefore not generalize to circumstances in which the model makes an incorrect prediction of risk or in which ED behaviors arise due to factors unmeasured in the planned data collection.

## Supplementary material

10.2196/104849 Multimedia Appendix 1 Sample just-in-time adaptive intervention (JITAI) content.

10.2196/104849 Peer Review Report 1 Peer review report by the ZRG1 BP-M (70) - Center for Scientific Review Special Emphasis Panel RFA Panel: NIH Director's Early Independence Award Review (National Institutes of Health, USA).

## Abbreviations

AUROC: area under the receiver operating characteristic curve
C-SSRS: Columbia-Suicide Severity Rating Scale
CBT: cognitive behavioral therapy
CBT-E: enhanced cognitive-behavioral therapy
ED: eating disorder
EDDI: Eating Disorder Dynamic Intervention
EDE: Eating Disorder Examination
EMA: ecological momentary assessment
HIPAA: Health Insurance Portability and Accountability Act
IRB: Institutional Review Board
JITAI: just-in-time adaptive intervention
NARMAX: nonlinear autoregressive moving average model with exogenous inputs
