# Supplementary material for: Rapid Innovation of Precision Psychiatry Interventions Using Dynamical Systems Modeling and Ecological Quasi-Experiments: Protocol for a Proof-of-Concept Study on Eating Disorders
Source: JMIR Res Protoc. 2026 Oct 15;15:e104849. doi: 10.2196/104849 (PMC13671667; doi:10.2196/104849)
Supplement: Supplementary file 1 [file resprot-v15-e104849-s002.pdf]

SUMMARY STATEMENT
(Privileged Communication)

PROGRAM CONTACT:
Becky Miller
301-594-9979
becky.miller2@nih.gov

Release Date: **04/29/2025**
Revised Date:

Application Number: **1DP5OD039510-01**

Principal Investigator

PRESSELLER, EMILY KELLEY

Applicant Organization: DREXEL UNIVERSITY

Review Group:	**ZRG1 BP-M (70)**<br>**Center for Scientific Review Special Emphasis Panel**<br>**RFA Panel: NIH Director's Early Independence Award Review**
Meeting Date:	**04/07/2025**
Council:	**MAY 2025**
Requested Start:	**09/01/2025**
Opportunity Number:	**RFA-RM-24-005**
Dual IC(s):	**RM**
Project Title:	**Rapid innovation of precision psychiatry interventions using dynamic systems modeling and ecological quasi-experiments**
SRG Action:	**Impact Score:27**
Next Steps:	**Visit https://grants.nih.gov/grants/next_steps.htm**
**Human Subjects:**	**48-At time of award, restrictions will apply**
**Animal Subjects:**	**10-No live vertebrate animals involved for competing appl.**
**Gender:**	**1A-Both sexes, scientifically acceptable**
**Minority:**	**1A-Minorities and non-minorities, scientifically acceptable**
**Age:**	**3A-No children included, scientifically acceptable**

Project Year	Direct Costs Requested	Estimated Total Cost
1	250,000	378,270
2	250,000	378,270
3	250,000	378,270
4	250,000	378,270
5	250,000	378,270
TOTAL	1,250,000	1,891,352

EARLY STAGE INVESTIGATOR
NEW INVESTIGATOR

1DP5OD039510-01 Presseller, Emily

EARLY STAGE INVESTIGATOR
NEW INVESTIGATOR
PROTECTION OF HUMAN SUBJECTS UNACCEPTABLE

RESUME AND SUMMARY OF DISCUSSION: This application proposes to improve the efficacy of precision psychiatry digital health interventions by developing real-world, person-centered maintenance models for psychopathology using ecological momentary assessment (EMA) data and dynamic systems modeling develop a just-in-time adaptive intervention (JITAI) to deliver micro-randomized therapeutic strategies at times of elevated risk for maladaptive eating disorder behaviors. During the discussion, the Editorial Board agreed that the investigator presented a strong rationale for independence, who had advanced training in statistical methods and EMA, identified collaborations outside of her university, and has a strong record of publications, funding, awards and supervision of graduate students that included research products. The Board considered the institutional environment to be strong for eating disorders and mHealth research, and the institutional support provided good salary support, protected time and the Board saw strong commitment to the investigator, but some members noted the amount of startup seemed modest and they were unsure if it was adequate for project needs. The Board found the this to be an innovative take on precision psychiatry and the target population of those with eating disorders warranted new approaches, making the project both innovative and significant. There were some concerns raised by the mail reviewers whether the approach was innovative enough to allow the candidate to move out of the shadow of former mentors, particularly as she is staying at the same institution, but the Board noted that the application focused on a broader range of eating disorders compared to prior mentors and that she has demonstrated independent thinking from her mentors which assuaged concerns. There was one disagreement between Board members if any ethical issues presented from delaying treatment to collect data in underweight individuals or those with significant symptomatology, but others were less concerned as participants this design would provide treatment as usual. Overall, the Board determined that the preparedness of the investigator, the institutional support, and the research plan would facilitate the candidate’s success and concluded that this Early Independence Award would have a high likelihood of accelerating the investigator’s research career.

DESCRIPTION (provided by applicant): The US faces a mental health crisis; nearly 83 million Americans experience mental illness, but fewer than 50% of these individuals accessed past year mental health services. There is a critical need for highly disseminable, potent mental health interventions. Standalone digital interventions offer promise for improving clinical outcomes at scale; however, efficacy of these interventions to date is modest, likely due in part to insufficient personalization to patient heterogeneity and to momentary changes in mental health symptoms. The present study seeks to rapidly improve the efficacy of precision psychiatry digital health interventions by developing real-world, person-centered maintenance models for psychopathology using ecological (i.e., in patient’s daily lives) data and dynamic systems modeling. Dynamic systems modeling of repeated time series data yielded by ecological momentary assessment (EMA) and smartphone sensors will be used to evaluate the individual and interactive effects of empirical maintenance factors to understand each individual’s maintenance system for psychopathology in their daily life. Just-in-time adaptive interventions (JITAIs) are prompts delivered in-the-moment via smartphone at identified instances of risk for maladaptive behaviors. Although often evaluated in aggregate across months, JITAIs could be conceptualized as ecological quasi-experiments that induce use of specific therapeutic skills at observable times. Accordingly, this study will use micro-randomized (in which the content of the JITAI is randomly assigned in the moment) JITAIs delivered at times of elevated risk for maladaptive behavior based on the individual’s developed dynamic system to evaluate the effects and mechanisms of

specific therapeutic skills on outcomes of interest, to provide granular insight into treatment mechanisms. Eating disorders are the ideal population in which to test proof-of-concept for these methods to improve efficacy of precision psychiatry digital interventions, as they are characterized by easily measurable maladaptive behaviors (i.e., dietary restriction, binge eating, compensatory behaviors), are maintained by a complex intersection of biological, psychology, and social factors, and their frontline treatment, enhanced cognitive behavioral therapy (CBT-E), is primarily comprised of behavioral skills. The aims are: 1) test the hypothesis that person-centered dynamic models will describe risk for dietary restriction, binge eating, add compensatory behaviors with ≥ 60% average goodness-of-fit, 2) test hypothesized effects and mechanisms of CBT-E skills on eating disorder behaviors and primary maintenance factors using JITAIs, and 3) characterize feasibility and acceptability of the standalone JITAIs. The study will enroll N = 170 adults with eating disorders who will complete four months of ecological data collection and receive JITAIs to use CBT-E skills at times of elevated risk for 10 weeks of the data collection period. The study will set the stage for future randomized controlled trail to evaluate the optimized, standalone JITAI system, which will have the potential to substantially improve access to evidence-based mental healthcare.

PUBLIC HEALTH RELEVANCE: There is an urgent need to develop effective and highly disseminable digital interventions to address the current mental health crisis in America. The present study will: 1) use ecological data collection and dynamic systems modeling to develop comprehensive, personalized risk models for eating disorder behaviors to inform the delivery of just-in-time adaptive interventions at times when individual patients are at risk for these behaviors, and 2) deliver micro-randomized just-in-time adaptive interventions at identified moments of risk to facilitate real-world testing of the effects and mechanisms of core therapeutic skills in an ecologically valid way. This study will have implications for future precision psychiatry efforts to personalize treatment for individual patients and digital interventions designed to deliver the right type of support to disrupt systems maintaining psychopathology, and yield an optimized, standalone digital intervention for eating disorders.

CRITIQUE 1

Significance: 1
Investigator(s): 1
Innovation: 1
Approach: 2
Environment: 2

Overall Impact: This DP5 application comes from a 5th-year clinical psychology doctoral student requesting 5 years of funding to establish an independent eating disorders research program as a newly appointed Assistant Research Professor at Drexel University. Her proposed study will enroll 170 adults (ages 18-70yo) with eating disorder symptoms to complete four months of ecological data collection (including both ecological momentary assessment and passive sensing). During the first month, the candidate will use dynamic systems modeling to develop idiographic models of predictors of symptom engagement. During months 2-4, she will use these models to deliver just-in-time-adaptive interventions (comprising individual cognitive-behavior therapy skills) at high-risk times in a micro-randomized design to test their efficacy and mediators. The candidate herself is exceptional, having already established a strong track record of publications, funding, awards, and teaching/mentoring experience that make her well suited to forgo post-doctoral training and start her own lab. Her project tackles a significant problem (lack of access to eating disorder treatments, low efficacy of current digital interventions) and uses innovative methods (passive sensing, dynamical systems modeling, machine learning), and is anchored to established theory (cognitive-behavioral model of eating disorders). The

environment is outstanding as it will provide both material resources (i.e., lab space, research assistant support, startup funding) and mentorship/consultation from senior faculty. A minor weakness of the environment is that the candidate plans to stay at the same institution where they trained as both a research coordinator and graduate student, which may slightly impede her ability to emerge as an independent faculty member. However, this is mitigated by her plan to collaborate with different mentors/faculty than those she'd worked with previously. Moderate weaknesses to the approach include the potential dependence of Aim 2 on the success of Aim 1 (i.e., if the idiographic models are a poor fit to the data, it is unclear how the JITAIs will be successfully deployed), and the tautological nature of sub-hypotheses in Aim 2 (i.e., that interventions designed to reduce dietary restriction will indeed reduce dietary restriction). That said, these modest weaknesses in the approach are far outweighed by the major strengths of the candidate and environment, and the highly innovative nature of the project. Overall, this proposal as strong potential for high impact on the field of eating disorders specifically and—potentially—mental health more broadly.

1. Significance:

Strengths

- The project focuses on an extremely important public health problem. Eating disorders have a high mortality rate, many individuals never receive treatment, and even evidence-based treatments for ED (e.g., cognitive-behavioral therapy).
- Technology-based interventions offer substantial promise for improving EDI outcomes and increasing access to supports among underserved populations.
- Increasing the precision of digital health tools has great potential to improve the efficacy and uptake of m-health tools, as they can be tailored to the specific circumstances maintaining psychopathology, and patients perceive them to be appropriate, timely, and useful.

Weaknesses

- None noted by reviewer.

2. Investigator(s):

Strengths

- This applicant is an outstanding candidate for a DP5 award.
- Multiple letters of recommendation from senior faculty describe her as among the top 1-2 junior colleagues they had ever mentored.
- She has an established track record of funding, with five early-career research grants (totaling > $100K in funding) including a NIMH F31 that earned a perfect score.
- Through her F31, she has already gained many of the skills she will need in ecological momentary assessment, sensors, dynamic modeling, and machine learning.
- She has published 31 peer-reviewed articles (16 first author) and given 44 conference presentations (20 first-author).
- She has won 10 awards for her research, including not just internal awards from Drexel but also those from professional societies (e.g., Academy for Eating Disorders).
- She has extensive mentoring experience for someone so early in her career (i.e., 7 undergraduates, 11 research coordinators, 5 junior graduate students).
- She is a sought-after consultant on technology-based interventions and passive sensing from faculty around the country.

Weaknesses

- None noted by reviewer.

3. Innovation:

Strengths

- The proposed study is extremely innovative. It will be the first to use dynamic modeling to develop idiographic models of ED symptom maintenance, and the first to use micro-randomization to experimentally test the impacts of CBT skills on ED symptoms and maintenance mechanisms.
- The use of novel technologies (passive sensing, JITAIs delivered via app), and novel data analytic approaches (machine learning) is exciting.
- The application of concepts from engineering (e.g., dynamic modeling) to mental health is very creative, and highlights the candidate’s strong potential for designing innovative research studies as she continues in her career.

Weaknesses

- None noted by reviewer.

4. Approach:

Strengths

- The logic behind the project is very compelling. The use of dynamic modeling provides an exciting opportunity to not only test the CBT theory of ED maintenance but also deploy JITAIs at optimal moments in the participants’ daily lives.
- The candidate presents compelling preliminary data (even though such data are not required by the DP5 mechanism). Specifically, the candidate’s prior studies support that (1) she can use machine learning models to predict momentary engagement in at least some ED behaviors (e.g., binge eating), and that (2) patients with ED perceive JITAI prompts to be feasible and helpful.
- The candidate’s established collaboration with Knowledge Excel (a technology company) increases confidence that she will be able to successfully develop an app to deliver JITAIs.

Weaknesses

- A minor weakness is that Aim 2 is somewhat dependent on Aim 1. Specifically, if the idiographic models do not adequately predict symptom use, it is unclear how the proposed JITAIs will successfully be deployed. That said, this weakness is somewhat mitigated as she would still be able to evaluate the efficacy of the individual JITAIs (just not the added benefit of them being ideographically deployed, if Aim 1 does not work).
- Another minor weakness is the lack of clarity around inclusion criteria. The study will include adults with at least 12 ED behaviors (i.e., binge eating, compensatory behaviors, dietary restriction) in the past 12 weeks. However, it is unclear whether 12 instances of dietary restriction would qualify the eating disorder. If some participants do not actually have full syndrome eating disorders, the public health impact of the findings may be reduced.
- A final minor weakness is the unclear utility of the planned manipulation check, in which participants will be asked 15 minutes after the prompt whether they engaged in the JITAI. The manipulation check could work well for certain interventions (e.g., set a timer for 15 mins to urge

surf) but for others (e.g., avoid body checking for the rest of the day) it gives insufficient time for the participant to use the intervention.

5. Environment:

Strengths

- The candidate has chosen an excellent environment for launching a DP5. Drexel is an R1 University, and the WELL Center in which the candidate will be embedded has 6 senior faculty who have sustained NIH funding and hold approximately $30 million in current funding including several R01s.
- In addition to start-up funds provided by the University, the WELL Center will provide the candidate with a generous startup package including direct funding ($20,000), a 50% FTE research coordinator, access to an administrative support professional, and 500 square feet of lab space.
- Dr. Meghan Butryn and Dr. Rose Ann DiMaria-Ghalili will provide professional development mentorship to the candidate in establishing her faculty career but will not be directly involved with execution of the research study (thus allowing the candidate to become independent).
- The WELL Center has an established recruitment pipeline for patients with eating disorders, which will facilitate completion of the study.

Weaknesses

- A minor weakness of the environment is that the candidate plans to stay at the same institution where she trained as both a research coordinator and graduate student. This may slightly impede her ability to emerge as an independent faculty member (given that many people may still think of her as junior). It also may limit her exposure to new perspectives. However, this minor weakness is mitigated by her plan to collaborate with different mentors/faculty than those who had previously mentored her.

Study Timeline

Strengths

- The timeline allows for a realistic 6-month period for lab set-up and study start-up before starting data collection.

Weaknesses

- The plan to develop the R01 proposal in Year 5 does not allow sufficient time for the applicant to maintain continuous funding once DP5 concludes, even if the R01 is funded on the first submission. Development of the R01 proposal should begin in Year 4 at the latest.
- The applicant plans for publication development in Year 5 only. It’s unclear whether there are plans to submit publications (perhaps on other data) in Years 1-4 to maintain consistent productivity for career development.

Protections for Human Subjects:

Acceptable Risks and/or Adequate Protections.

- Compensation scheme for participants seems punitive (i.e., subtracting $2/day from advertised compensation rate for EMA compliance < 70%; would suggest instead advertising a lower base compensation rate, with the possibility of earning $2/day bonus for compliance)

Data and Safety Monitoring Plan (Applicable for Clinical Trials Only):

Acceptable

- Comprehensive plan identifies expert independent safety officer (Dr. Boswell), describes process for adverse event reporting, and reasonable stopping rules.

Inclusion Plans:

- Sex: Distribution justified scientifically
- Race/Ethnicity: Distribution justified scientifically
- For NIH-Defined Phase III trials, Plans for valid design and analysis: Not applicable
- Inclusion/Exclusion Based on Age: Distribution justified scientifically
- Recruiting males and females ages 18-70 years old, scientifically appropriate b/c testing adult CBT model of ED pathology; efforts will be made to recruit a sample in which 50% are from racial/ethnic minoritized groups; data analysis plan is appropriate

Vertebrate Animals:

Not Applicable (No Vertebrate Animals)

Biohazards:

Not Applicable (No Biohazards)

Resource Sharing Plans:

Acceptable

- Will register on clinicaltrials.gov and share data on NIMH National Data Archive

Budget and Period of Support:

Recommend as Requested.

CRITIQUE 2

Significance: 3
Investigator(s): 1
Innovation: 2
Approach: 5
Environment: 3

Overall Impact: This NIH Director's Early Independence Award (DP5) application aims to improve the efficacy of digital standalone just-in-time adaptive interventions for mental health (JITAIs) by 1) developing idiographic (i.e., person-specific) models of eating disorder behavior risk using dynamic systems modeling to analyze ecological momentary assessment (EMA) and smartphone sensor data; and 2) experimentally testing the effects and mechanisms of therapeutic skills on disordered eating behaviors using micro-randomized JITAIs. The PI's rationale for early independence is well-justified

based on her record of successfully executing studies using similar data collection and analytic methods, productivity in publishing manuscripts (31 total; 16 as first author), mentorship of research staff and more junior trainees, and collaborations with investigators outside of her department. Moreover, the environment in the WELL Center at Drexel University is ideally suited to establishing an independent career conducting research on mHealth and behavioral interventions for eating disorders, and there is a strong commitment to the PI. Additional score-driving strengths of the application include the significance of using precision psychiatry methods to enhance the efficacy of digital mental health interventions, especially for individuals with eating disorders who often do not receive specialized treatment; the innovative application of dynamic systems modeling (an approach from engineering) to build idiographic models of eating disorder behavior risk, as well as the innovative use of micro-randomized JITAIs to experimentally test risk and maintenance mechanisms for eating disorder behaviors in the natural environment; and the compelling logic of the approach, which builds directly from the PI's prior research experiences. Detracting from the application's strengths, some moderate weaknesses were noted in the approach that raise concern about the environment's ability to support the PI's research incorporating the full spectrum of eating disorders. Specifically, ethical issues involved in the management of high-risk eating disorder presentations (e.g., restrictive eating disorders, purging) are not fully considered, including plans for monitoring symptom worsening and the rationale delaying delivery of JITAIs in a sample where eating disorder-focused treatment is an exclusion criterion. Nevertheless, the application's strengths outweigh its weaknesses, and overall impact is expected to be high.

1. Significance:

Strengths

- The proposed project addresses a critical need to enhance the efficacy of digital mental health interventions, given their scalability and the fact that many patients do not access traditional mental health services.
- Precision psychiatry is a promising approach to improving the efficacy of digital tools, but effect sizes associated with existing treatment personalization efforts are small. If successful, the proposed project will increase the potency of precision psychiatry digital tools, which would have a significant impact on mental health outcomes.
- Eating disorders are an important population in which to test new digital interventions, given that they often are untreated and can result in serious medical and psychiatry morbidity, as well as increased mortality.
- Results of the proposed project will have relevance to the design of digital tools to address non-eating disorder-related mental health outcomes.

Weaknesses

- One month of data is required to build the ideographic maintenance models prior to the delivery of just-in-time adaptive interventions. Delaying intervention for this period may not be realistic or ethical in some populations (e.g., underweight individuals; those with significant purging).

2. Investigator(s):

Strengths

- The PI's rationale for early independence is well-justified based on her record of successfully executing and publishing original research.

- The PI’s skills in EMA and sensor data collection, user centered design and mHealth interventions, advanced statistical methods (including machine learning), and treatment of eating disorders place her in an excellent position to conduct highly innovative research.
- The PI has experience collaborating with investigators outside of her institution and has been asked to consult on the design of studies using EMA and sensor technology.
- The PI has mentored 7 undergraduates, 11 research coordinators, and 5 junior graduate students, resulting in 8 manuscripts, 9 presentations, and 6 federal research grants.
- The Early Independence Award would accelerate the PI’s transition to scientific independence by allowing her to extend her current skillset and program of research rather than working on other projects.
- There is a strong likelihood that this award will result in publications and grant applications that would benefit the PI’s long-term career.
- Letters of reference are very strong and indicate that the PI is ready for an independent scientific career.

Weaknesses

- No weaknesses noted.

3. Innovation:

Strengths

- The use of dynamic systems modeling (an approach from engineering) to build ideographic models of risk for eating disorder behaviors based on EMA and sensor data is highly innovative, as this approach has never been used in mental health research.
- Using micro-randomized just-in-time adaptive interventions as quasi-experimental manipulations to test ecological risk and maintenance mechanisms for eating disorder behaviors also is highly innovative and could advance models of eating disorder risk and maintenance.
- A digital intervention that adapts intervention criteria based on individual changes in other maintaining factors would be unique in the eating disorders field.

Weaknesses

- Basing a digital tool for eating disorders on CBT-E is not innovative, though this is a minor concern.

4. Approach:

Strengths

- The approach builds logically on the PI’s prior research experiences, incorporating novel methods (e.g., dynamic systems modeling) to address limitations the PI observed in work designing and testing digital tools for eating disorders.
- EMA and passive sensor data will be integrated in the dynamic systems models.
- The analytic strategy is well-reasoned and appropriate for testing the study hypotheses.

Weaknesses

- Just-in-time adaptive interventions (JITAIs) to address eating disorder symptoms will not be delivered until week 3 of month 2. This raises ethical concerns given that eating disorder-focused treatment is an exclusion criterion for the study.

- There are no plans for addressing symptom worsening over the course of the study, and it is unclear how the accuracy of exclusion criteria related to body weight, and medical complications will be verified.
- There may be diagnostic differences in participant willingness to use JITAIs (e.g., greater ambivalence in participants with anorexia nervosa), and it is unclear how this will be addressed in the analyses.
- The project scope is ambitious in terms of recruitment rate and management of a heterogeneous eating disorder sample.
- It is unclear why EDE interviews will only be administered at baseline (versus at baseline and the end-of-study visit).

5. Environment:

Strengths

- The PI's lab will be housed in the WELL Center at Drexel University, an exceptional environment for conducting research on mHealth and behavioral interventions for eating disorders. She will have office space for herself and her staff/trainees (4 total offices) and access to shared conferences rooms, assessment spaces, and support staff.
- The PI will be appointed as an Assistant Research Professor in the WELL Center, starting September 1, 2025. This appointment is not contingent on receipt of the DP5 Award.
- Drexel University and the WELL Center are committed to protecting at least 80% of the PI's time for independent research and will provide startup funding and access to internal grants for pilot research.
- Plans are in place to ensure the PI's scientific independence from previous mentors.
- Several strategies for addressing potential problems are described including university trainings and courses and mentorship from faculty the PI has not worked with previously.

Weaknesses

- The PI will distinguish herself from previous mentors and other faculty, in part, by focusing on a broader range of eating disorders (i.e., anorexia nervosa [AN] and atypical AN, in addition to binge spectrum eating disorders). However, there are unique challenges involved in designing interventions for restrictive eating disorders, and it is unclear if the environment will provide adequate support for this work.

Study Timeline

Strengths

- The timeline is very detailed and includes 6 months in Year 1 for startup and 6 months in Year 5 for data analysis and publication.

Weaknesses

- Recruitment of 170 participants in 42 months is ambitious, and strategies that will be implemented in the event of recruitment shortfalls are not described.

Protections for Human Subjects:

Unacceptable Risks and/or Inadequate Protections

- The protocol for addressing the likely possibility that some participants' eating disorder symptoms will worsen over the course of the study is unclear. The DSMP mentions "continual safety monitoring," but does not specify whether this will be done systematically (by monitoring digital data) or only if a participant alerts the team to worsening symptoms. It also is unclear how safety will be ensured for participants outside of the Philadelphia region.

Data and Safety Monitoring Plan (Applicable for Clinical Trials Only):

Acceptable

- An independent safety monitor with expertise in the treatment of eating disorders will oversee the study

Inclusion Plans:

- Sex: Distribution justified scientifically
- Race/Ethnicity: Distribution justified scientifically
- For NIH-Defined Phase III trials, Plans for valid design and analysis: Not applicable
- Inclusion/Exclusion Based on Age: Distribution justified scientifically
- Women and men aged 18-70 years will be recruited in a 2:1 ratio, reflecting gender differences in eating disorder prevalence. Racial/ethnic minorities will comprise 50% of the sample.

Vertebrate Animals:

Not Applicable (No Vertebrate Animals)

Biohazards:

Not Applicable (No Biohazards)

Resource Sharing Plans:

Acceptable

Budget and Period of Support:

Recommend as Requested.

CRITIQUE 3

Significance: 4
Investigator(s): 2
Innovation: 5
Approach: 5
Environment: 1

Overall Impact: This Early Independence Award application seeks to develop personalized risk models for eating disordered behaviors, with a view to developing just-in-time interventions to modify risk factors. The central premise is that eating disorder risk can be characterized and modeled based on

ecological momentary assessment data, and moreover, that these models can inform the development of smartphone driven CBT prompts to patients. Score-driving strengths include the candidate's history of high research productivity, the support of a highly productive institution, and the likely significance of this research area. Enthusiasm is tempered by some missing details relating to the methodological approach and relating to previous works relating to both the construction of idiographic models of ED psychopathology, and JITAI interventions and smartphone-based CBT-E prompts for EDs.

1. Significance:

Strengths

- Eating disorders (ED) are complex psychiatric disorders which carry an elevated risk for mortality, morbidity, and chronicity.
- EDs are a good target for JITAI interventions, given the dynamically changing and individualized context which gives rise to eating disorder symptoms.
- Treatment outcomes for eating disorders have seemingly reached a plateau, and the need for scalable interventions which target individual level symptoms cannot be overstated.

Weaknesses

- While noting that effect sizes for treatment personalization remain small, there is a paucity of data discussed to support the arguments made as to why this may be the case. This renders parts of the argument for the significance of the study speculative, rather than data driven.
- Some of the models noted in the significance section appear somewhat underdiscussed. For example, the notion that neurobiological signals attempt to restore weight homeostasis when a patient is underweight, is not discussed or elaborated upon, and these same neurobiological signals are seemingly not incorporated into the model.
- Previous work using JITAIs in the ED field are not comprehensively discussed. Since some of these existing studies stem from the same center, this evidence base ought to be comprehensively discussed, so it can be made clear how the proposed study builds on this previous work. Some of the studies presented as pilot data are discussed, although broader studies in the ED field developing idiographic models of ED maintenance, and use of CBT-E prompts, are not discussed.
- Some of the components in the proposed model are not discussed. For instance, it is not discussed how keyboard dynamics, call frequency, and location, are expected to relate to ED symptoms.

2. Investigator(s):

Strengths

- The candidate has an impressive body of published work, which relates directly to the nature of the proposed work. This work includes 31 peer-reviewed manuscripts, 16 of which are first-authored by the candidate.
- The candidate has published several manuscripts which included the methods proposed in the current application, enhancing project feasibility.
- Strong letters of support from highly impactful independent researchers in the field further attest to the candidate's potential impact on the ED field.

Weaknesses

- The candidate alludes to an array of previous training domains when justifying the decision made to omit postdoctoral training, all of which are relevant to the proposed work. However, details of this previous training are not provided in depth, making it somewhat difficult to ascertain the readiness for research independence.

3. Innovation:

Strengths

- The development of scalable, optimized JITAIs is an important goal in the field of eating disorders.

Weaknesses

- Some aspects of the purported innovation appear overstated. The use of ecological momentary assessment (inclusive of smartphone sensor data) to develop personalized, idiographic models of eating disorder psychopathology is not inherently novel and has been well explicated across a range of eating disorder psychopathologies.
- Smartphone driven CBT-E prompts are not inherently novel in the treatment of eating disorders.
- JITAIs have been investigated in the context of eating disorders, and a recent RCT was conducted by the same center where the candidate is based. It is unclear how the proposed work builds on these recent works.

4. Approach:

Strengths

- The inclusion of a large sample size, and the inclusion of males with eating disorders, is a methodological strength.
- The inclusion of a clinician-administered interview for ED psychopathology is a strength.
- The candidate presents preliminary data relating to the proposed work.

Weaknesses

- Recruitment will be undertaken nationally via online sampling methods, but it is unclear how, or whether, ED diagnoses will be confirmed. While appreciating that the analyses are intended to be dimensional, the broader rationale for the proposal appears to be related to enhancing the scalability of clinical treatments for EDs. As such, it would enhance the impact and scalability of this work if it were possible to ascertain the effect of the proposed JITAI in the clinical populations alluded to in outlining the project rationale.
- Relatedly, it is unclear who will be conducting the EDE, and whether EDE training has been undertaken.
- Medical complications of EDs are listed as an exclusionary criterion, but it is unclear how this will be addressed, and by whom.
- The presence of severe psychopathology is listed as an exclusion criterion, but it is unclear how this will be screened for and assessed.
- The statistical analysis plan appears underdeveloped, particularly for Aim 3.
- Push notifications are to be delivered when there is a 50% risk of engaging in ED behaviors, but it is unclear how this risk score will be determined, and what this factors in.
- Micro-randomization is listed as an innovation, but how this is undertaken is not explicated.

5. Environment:

Strengths

- Strong letters of institutional support are evident, highlighting the resources available for this project.
- The WELL Center is a remarkably productive center, with a strong track record of grant funding and high impact research.

Weaknesses

- The position offered to Ms. Presseller is not a permanent or tenure track position at Drexel, limiting the institutional support for a period of 12 months. The continuity of institutional support, beyond this initial period, is unclear.

Study Timeline

Strengths

- Appropriate.

Weaknesses

- No weaknesses noted.

Protections for Human Subjects:

Acceptable Risks and/or Adequate Protections

- Appropriate.

Data and Safety Monitoring Plan (Applicable for Clinical Trials Only):

Acceptable

- Appropriate

Inclusion Plans:

- Sex: Distribution justified scientifically
- Race/Ethnicity: Distribution justified scientifically
- For NIH-Defined Phase III trials, Plans for valid design and analysis: Not applicable
- Inclusion/Exclusion Based on Age: Distribution justified scientifically
- Appropriate

Vertebrate Animals:

Not Applicable (No Vertebrate Animals)

Biohazards:

Not Applicable (No Biohazards)

Resource Sharing Plans:

Acceptable

- Appropriate

Authentication of Key Biological and/or Chemical Resources:

Not Applicable (No Relevant Resources)

Budget and Period of Support:

Recommend as Requested.

- Appropriate

CRITIQUE 4

Significance: 3
Investigator(s): 1
Innovation: 2
Approach: 6
Environment: 3

Overall Impact: This is a very novel and innovative project submitted by a highly accomplished PI who is just finishing up her clinical psychology PhD. Overall, the candidate is extremely impressive, and I expect that she has a bright future ahead of her. Her project is very interesting and significant. We need better digital treatments for psychopathology and in particular for eating disorders. One primary way to improve treatments is through personalization. Thus, the topic and significance and the PIs ideas are exactly the type of work that is in need of support. I have concerns about the project idea and approach, primarily because the scope of work presented does not seem feasible in the time span of five years and under this mechanism and without additional training. While the PI has significant experience with digital technologies and development and has a strong quantitative background, she is attempting to learn and apply dynamic systems theory and idiographic modeling to then apply just-in-time-adaptive interventions (JITAI) using passive data. There are few details in the proposal on how she will learn dynamic systems modeling, how it will be applied and how the data will be processed idiographically. These types of models are extremely complex and take a significant amount of type to learn and apply as idiographic models, even without the addition of passive data. This paired with then delivering JITAIs to test the impact of each intervention on specific target mechanisms is an enormous amount of training and work. I think this line of research is extremely important and needed, but I am not convinced the feasibility is currently there. As such, I think this would have moderate impact on the field.

1. Significance:

Strengths

- Combination of EMA and passive data is novel and exciting.
- Personalization of treatment can improve outcomes.
- Identifying if components of CBT move target symptoms is extremely significant and important.

Weaknesses

- There is a large amount of literature on personalization, both in terms of eating disorders and other forms of psychopathology that are missing that could inform the project.
- CBT only works for 50% of people even in standardized 'full dose' form.

2. Investigator(s):

Strengths

- The investigator is extremely strong, with 31 publications (16 first author) to date.
- She successfully completed an F31.
- PI has extensive training in novel methods, including sensor technology and mHealth techniques.

Weaknesses

- None noted by reviewer.

3. Innovation:

Strengths

- Combination of EMA, sensors is novel.
- Use of JITAI as quasi-experiment is very innovative.

Weaknesses

- None noted by reviewer.

4. Approach:

Strengths

- Idiographic modeling is a strength.
- Use of passive data is a strength.
- Well thought out matching of treatments to symptom.

Weaknesses

- No plan for processing passive data.
- Lack of information on dynamic systems modeling and how these data will be modeled.
- More justification for concentration on specific CBT skills could be provided.
- No plans for what to do if idiographic models do not converge in the way that is hoped/expected.

5. Environment:

Strengths

- Strong environment with many collaborators.
- Consultants are strong and have expertise in needed areas. (dynamical systems, engineering, behavior change)

Weaknesses

- PI has been at the same institute for most of her career.

Study Timeline

Strengths

- None noted by reviewer.

Weaknesses

- Not enough time allotted for data analysis or for how to integrate dynamic systems modeling into plan.

Protections for Human Subjects:

Acceptable Risks and/or Adequate Protections

Data and Safety Monitoring Plan (Applicable for Clinical Trials Only):

Acceptable

Inclusion Plans:

- Sex: Distribution justified scientifically
- Race/Ethnicity: Distribution justified scientifically
- For NIH-Defined Phase III trials, Plans for valid design and analysis: Not applicable
- Inclusion/Exclusion Based on Age: None noted by reviewer

Vertebrate Animals:

Not Applicable (No Vertebrate Animals)

Biohazards:

Not Applicable (No Biohazards)

Resource Sharing Plans:

Acceptable

Budget and Period of Support:

Recommend as Requested.

THE FOLLOWING SECTIONS WERE PREPARED BY THE SCIENTIFIC REVIEW OFFICER TO SUMMARIZE THE OUTCOME OF DISCUSSIONS OF THE REVIEW COMMITTEE, OR REVIEWERS' WRITTEN CRITIQUES, ON THE FOLLOWING ISSUES:

PROTECTION OF HUMAN SUBJECTS: UNACCEPTABLE

Reviewers raised concerns that the protocol for addressing worsening of eating disorder symptoms was insufficiently clear, and it was also unclear how the safety of participants outside of the Philadelphia area would be ensured; however, the Editorial Board were less concerned about delays in treatment due to participants' receiving treatment as usual.

INCLUSION OF WOMEN PLAN: ACCEPTABLE

INCLUSION OF MINORITIES PLAN: ACCEPTABLE

INCLUSION ACROSS THE LIFESPAN PLAN: ACCEPTABLE

COMMITTEE BUDGET RECOMMENDATIONS: The budget was recommended as requested.

Footnotes for 1DP5OD039510-01; PI Name: Presseller, Emily Kelley

NIH has modified its policy regarding the receipt of resubmissions (amended applications).See Guide Notice NOT-OD-18-197 at https://grants.nih.gov/grants/guide/notice-files/NOT-OD-18-197.html. The impact/priority score is calculated after discussion of an application by averaging the overall scores (1-9) given by all voting reviewers on the committee and multiplying by 10. The criterion scores are submitted prior to the meeting by the individual reviewers assigned to an application, and are not discussed specifically at the review meeting or calculated into the overall impact score. Some applications also receive a percentile ranking. For details on the review process, see http://grants.nih.gov/grants/peer_review_process.htm#scoring.

MEETING ROSTER

Center for Scientific Review Special Emphasis Panel
CENTER FOR SCIENTIFIC REVIEW
RFA Panel: NIH Director's Early Independence Award Review

ZRG1 BP-M (70)
04/07/2025 - 04/08/2025

Notice of NIH Policy to All Applicants: Meeting rosters are provided for information purposes only. Applicant investigators and institutional officials must not communicate directly with study section members about an application before or after the review. Failure to observe this policy will create a serious breach of integrity in the peer review process, and may lead to actions outlined in NOT-OD-22-044 at https://grants.nih.gov/grants/guide/notice-files/NOT-OD-22-044.html, including removal of the application from immediate review.

CHAIRPERSON(S)

HOGQUIST, KRISTIN A, PHD
PROFESSOR
DEPARTMENT OF LABORATORY MEDICINE AND PATHOLOGY
UNIVERSITY OF MINNESOTA
MINNEAPOLIS, MN 55455

MEMBERS

BANGASSER, DEBRA A, PHD
PROFESSOR
DEPARTMENT OF NEUROSCIENCE
COLLEGE OF ARTS & SCIENCE
GEORGIA STATE UNIVERSITY
ATLANTA, GA 30303

BRASIER, ALLAN R, MD
PROFESSOR
DEPARTMENT OF INTERNAL MEDICINE
UNIVERSITY OF WISCONSIN-MADISON
MADISON, WI 53597

DAVIES, STELLA M, PHD
PROFESSOR AND JACOB G. SCHMIDLAPP ENDOWED CHAIR
DIVISION OF EXPERIMENTAL HEMATOLOGY
CINCINNATI CHILDREN'S HOSPITAL MEDICAL CENTER
CINCINNATI, OH 45229

GEWIRTZ, DAVID A, PHD
PROFESSOR
DEPARTMENT OF PHARMACOLOGY AND TOXICOLOGY
VIRGINIA COMMONWEALTH UNIVERSITY
RICHMOND, VA 23298

HENNESSY, SEAN, PHARMD, PHD
PROFESSOR OF EPIDEMIOLOGY, SYSTEMS PHARMACOLOGY AND TRANSLATIONAL THERAPEUTICS
CENTER FOR PHARMACOEPIDEMIOLOGY RESEARCH AND TRAINING
PERELMAN SCHOOL OF MEDICINE
UNIVERSITY OF PENNSYLVANIA
PHILADELPHIA, PA 19104

HUNT, PATRICIA, PHD
PROFESSOR
SCHOOL OF MOLECULAR BIOSCIENCES
WASHINGTON STATE UNIVERSITY
PULLMAN, WA 99164

LAWRENCE, THEODORE S, PHD, MD
ISADORE LAMPE PROFESSOR AND CHAIR
DEPARTMENT OF RADIATION ONCOLOGY
UNIVERSITY OF MICHIGAN
ANN ARBOR, MI 48109

MCCULLOCH, ANDREW D, PHD
PROFESSOR
DEPARTMENT OF BIOENGINEERING AND MEDICINE
UNIVERSITY OF CAIFORNIA SAN DIEGO
LA JOLLA, CA 92093

OFILI, ELIZABETH O., MD
PROFESSOR OF MEDICINE
CLINICAL RESEARCH CENTER
MOREHOUSE SCHOOL OF MEDICINE
ATLANTA, GA 30310

ORTLUND, ERIC A, PHD
PROFESSOR AND VICE CHAIR
DEPARTMENT OF BIOCHEMISTRY
SCHOOL OF MEDICINE
EMORY UNIVERSITY
ATLANTA, GA 30322

WICHMAN, HOLLY A, PHD
DISTINGUISHED PROFESSOR AND DIRECTOR
DEPARTMENT OF BIOLOGICAL SCIENCES
INSTITUTE FOR MODELING COLLABORATION AND INNOVATION
UNIVERSITY OF IDAHO
MOSCOW, ID 83844

MAIL REVIEWER(S)

AARONSON, SCOTT T, MD
ADJUNCT PROFESSOR
DEPARTMENT OF PSYCHIATRY
UNIVERSITY OF MARYLAND SCHOOL OF MEDICINE
BALTIMORE, MD 21204

ALEXEEFF, STACEY E, PHD
RESEARCH SCIENTIST II BIOSTATISTICIAN
DIVISION OF RESEARCH
KAISER PERMANENTE NORTHERN CALIFORNIA
OAKLAND, CA 94612

ALGARIN, ANGEL B, PHD
ASSISTANT PROFESSOR
CENTER FOR HEALTH PROMOTION AND DISEASE PREVENTION
ARIZONA STATE UNIVERSITY
PHOENIX, AZ 85013

ALILAIN, WARREN J, PHD
ASSOCIATE PROFESSOR
DEPARTMENT OF SPINAL CORD AND
BRAIN INJURY RESEARCH CENTER
UNIVERSITY OF KENTUCKY
LEXINGTON, KY 40506

ALOSCO, MICHAEL, PHD
ASSOCIATE PROFESSOR
DEPARTMENT OF NEUROLOGY
BU CHOBANIAN & AVEDISIAN SCHOOL OF MEDICINE
BOSTON, MA 02118

AN, XIULI, MD, PHD
MEMBER
LABORATORY OF MEMBRANE BIOLOGY
LINDSLEY F. KIMBALL RESEARCH INSTITUTE
NEW YORK BLOOD CENTER
NEW YORK CITY, NY 10065

ANTO-OCRAH, MARTINA, PHD
ASSISTANT PROFESSOR
DIVISION OF GENERAL INTERNAL MEDICINE
DEPARTMENT OF MEDICINE
UNIVERSITY OF PITTSBURGH
PITTSBURGH, PA 15261

ARIAS, JALAYNE J, JD
ASSOCIATE PROFESSOR
DEPARTMENT OF HEALTH POLICY & BEHAVIORAL SCIENCES
GEORGIA STATE UNIVERSITY
140 DECATUR STREET
ATLANTA, GA 30302

ASCHEBROOK-KILFOY, BRISEIS A, PHD
PROFESSOR
BIOLOGIC SCIENCES DIVISION
DEPARTMENT OF PUBLIC HEALTH SCIENCES
INSTITUTE FOR POPULATION AND PRECISION HEALTH
THE UNIVERSITY OF CHICAGO
CHICAGO, IL 60637

BAGCHI, INDRANI C, PHD
ASSOCIATE PROFESSOR
DEPARTMENT OF VETERINARY BIOSCIENCE
UNIVERSITY OF ILLINOIS-URBANA
URBANA, IL 61802

BAI, WUBIN, PHD
ASSISTANT PROFESSOR
DEPARTMENT OF APPLIED PHYSICAL SCIENCES
UNIVERSITY OF NORTH CAROLINA AT CHAPEL HILL
CHAPEL HILL, NC 27599

BAILEY, STACY C, PHD
PROFESSOR
DIVISION OF GENERAL INTERNAL MEDICINE
FEINBERG SCHOOL OF MEDICINE
NORTHWESTERN UNIVERSITY
CHICAGO, IL 60611

BALDWIN, MATTHEW R, MD
COLUMBIA UNIVERSITY MEDICAL CENTER
NEW YORK, NY 10032

BARNETT, BRIAN S, MD
ASSISTANT PROFESSOR
DEPARTMENT OF PSYCHIATRY
CLEVELAND CLINIC
CLEVELAND, OH 44113

BASLER, CHRISTOPHER F, PHD
PROFESSOR
DEPARTMENT OF MICROBIOLOGY
ICAHN SCHOOL OF MEDICINE AT MOUNT SINAI
NEW YORK, NY 10029

BENICHOU, GILLES A, DSC, PHD
ASSOCIATE PROFESSOR
DEPARTMENT OF SURGERY, TRANSPLANTATION UNIT
MASSACHUSETTS GENERAL HOSPITAL
HARVARD MEDICAL SCHOOL
BOSTON, MA 02114

BERENFELD, OMER, PHD
PROFESSOR
DEPARTMENT OF INTERNAL MEDICINE AND
OF BIOMEDICAL ENGINEERING
CENTER FOR ARRHYTHMIA RESEARCH
UNIVERSITY OF MICHIGAN HEALTH SYSTEM
ANN ARBOR, MI 48109

BEZDUDNAYA, TATIANA, PHD
ASSISTANT PROFESSOR
DEPTARTMENT OF NEUROBIOLOGY AND ANATOMY
DREXEL UNIVERSITY COLLEGE OF MEDICINE
PHILADELPHIA, PA 19129

BICK, ALEXANDER, MD, PHD
ASSOCIATE PROFESSOR
DEPARTMENT OF MEDICINE
VANDERBILT UNIVERSITY SCHOOL OF MEDICINE
NASHVILLE, TN 37232

BIEKER, JAMES J, PHD
PROFESSOR
DEPARTMENT OF CELL, DEVELOPMENTAL AND
REGENERATIVE BIOLOGY
MOUNT SINAI SCHOOL OF MEDICINE
NEW YORK, NY 10029

BIELINSKI, SUZETTE J, PHD
PROFESSOR
DIVISION OF EPIDEMIOLOGY
MAYO CLINIC COLLEGE OF MEDICINE
ROCHESTER, MN 55905

BLACKSHAW, SETH, PHD
PROFESSOR
DEPARTMENT OF NEUROSCIENCE
SCHOOL OF MEDICINE
JOHNS HOPKINS UNIVERSITY
BALTIMORE, MD 21287

BRICENO, EMILY M, PHD
ASSOCIATE PROFESSOR
DEPARTMENT OF PHYSICAL MEDICINE AND
REHABILITATION
UNIVERSITY OF MICHIGAN
ANN ARBOR, MI 48108

BROMBERG, JONATHAN S, MD, PHD
PROFESSOR, VICE CHAIR
SURGERY, MICROBIOLOGY AND IMMUNOLOGY
CENTER FOR VASCULAR AND INFLAMMATORY DISEASES
UNIVERSITY OF MARYLAND
BALTIMORE, MD 21201

BURATOWSKI, STEPHEN, PHD
PROFESSOR
DEPARTMENT OF BIOLOGICAL CHEMISTRY
AND MOLECULAR PHARMACOLOGY
HARVARD MEDICAL SCHOOL
BOSTON, MA 02115

BURG, MATTHEW M, PHD
PROFESSOR
DEPARTMENT OF INTERNAL MEDICINE
YALE UNIVERSITY SCHOOL OF MEDICINE
NEW HAVEN, CT 06520

CALIFANO, JOSEPH A, MD
PROFESSOR AND VICE CHIEF OF DIVISION OF
OTOLARYNGOLOGY
DEPARTMENT OF SURGERY
DIVISION OF OTOLARYNGOLOGY
HEAD AND NECK CANCER CENTER
UNIVERSITY OF CALIFORNIA, SAN DIEGO
LA JOLLA, CA 92037

CANOLL, PETER, MD, PHD
PROFESSOR
DIRECTOR OF NEUROPATHOLOGY
DEPARTMENT OF PATHOLOGY AND
CELL BIOLOGY
COLUMBIA UNIVERSITY MEDICAL CENTER
NEW YORK, NY 10032

CHANNAPPANAVAR, RUDRAGOUDA, DVM, PHD
ASSISTANT PROFESSOR
DEPARTMENTS OF VIRAL IMMUNOLOGY AND
VETERINARY PATHOBIOLOGY
SCHOOL OF VETERINARY MEDICINE
OKLAHOMA STATE UNIVERSITY
STILLWATER, OK 74078

CHEN, BIN, PHD
ASSOCIATE PROFESSOR
DEPARTMENT OF PEDIATRICS AND HUMAN DEVELOPMENT
COLLEGE OF HUMAN MEDICINE
MICHIGAN STATE UNIVERSITY
EAST LANSING, MI 48824

CHEN, KAI, PHD
ASSISTANT PROFESSOR
DEPARTMENT OF ENVIRONMENTAL HEALTH SCIENCES
SCHOOL OF PUBLIC HEALTH
YALE UNIVERSITY
NEW HAVEN, CT 65208

CHEN, SIDI, PHD
ASSOCIATE PROFESSOR
DEPARTMENT OF GENETICS AND NEUROSURGERY
YALE UNIVERSITY
NEW HAVEN, CT 06520

CHENG, CHAO, PHD
ASSOCIATE PROFESSOR
DEPARTMENT OF MEDICINE
BAYLOR COLLEGE OF MEDICINE
HOUSTON, TX 77030

CHRASTIL, ELIZABETH, PHD
ASSOCIATE PROFESSOR
DEPARTMENT OF NEUROBIOLOGY AND BEHAVIOR
SCHOOL OF BIOLOGICAL SCIENCES
UNIVERSITY OF CALIFORNIA, IRVINE
IRVINE, CA 92697

CISLER, JOSHUA M, PHD
ASSOCIATE PROFESSOR
DEPARTMENT OF PSYCHIATRY AND BEHAVIORAL SCIENCES
DELL MEDICAL SCHOOL
THE UNIVERSITY OF TEXAS AT AUSTIN
AUSTIN, TX 78705

COLLERY, ROSS F, PHD
ASSOCIATE PROFESSOR
DEPARTMENT OF OPHTHALMOLOGY AND VISUAL SCIENCES
EYE INSTITUTE, MEDICAL COLLEGE OF WISCONSIN
MILWAUKEE, WI 53226

COON, DAVID W, PHD
ASSOCIATE DEAN FOR RESEARCH AND PROFESSOR
EDSON COLLEGE OF NURSING AND HEALTH INNOVATION
ARIZONA STATE UNIVERSITY
PHOENIX, AZ 85004

CUI, FENG, PHD
PROFESSOR
THOMAS H. GOSNELL SCHOOL OF LIFE SCIENCES
ROCHESTER INSTITUTE OF TECHNOLOGY
ROCHESTER, NY 14623

DA SILVA GOMES, ANA, PHD
ASSISTANT MEMBER
DEPARTMENT OF MOLECULAR ONCOLOGY
H. LEE MOFFITT CANCER CENTER
TAMPA, FL 33612

DANNEMILLER, KAREN, PHD
ASSOCIATE PROFESSOR
CIVIL, ENVIRONMENTAL & GEODETIC ENGINEERING
COLLEGE OF ENGINEERING
THE OHIO STATE UNIVERSITY
COLUMBUS, OH 43210

DEGREGORI, JAMES V, PHD
COURTENAY C. AND LUCY PATTEN DAVIS ENDOWED CHAIR
DEPARTMENT OF BIOCHEMISTRY
AND MOLECULAR GENETICS
ANSCHUTZ MEDICAL CAMPUS
UNIVERSITY OF COLORADO
AURORA, CO 80045

DEGTEREV, ALEXEI, PHD
ASSOCIATE PROFESSOR
DEPARTMENT OF DEVELOPMENTAL, MOLECULAR AND CHEMICAL BIOLOGY
TUFTS UNIVERSITY
BOSTON, MA 02111

DEN HARTIGH, LAURA J, PHD
RESEARCH ASSOCIATE PROFESSOR
DEPARTMENT OF MEDICINE
METABOLISM, ENDOCRINOLOGY, AND NUTRITION
UNIVERSITY OF WASHINGTON
SEATTLE, WA 98109

DOGRA, PRASHANT, PHD
RESEARCH SCIENTIST
MATHEMATICS IN MEDICINE PROGRAM
HOUSTON METHODIST RESEARCH INSTITUTE
HOUSTON, TX 77030

DRECHSLER, YVONNE, PHD
PROFESSOR
COLLEGE OF VETERINARY MEDICINE
WESTERN UNIVERSITY OF HEALTH SCIENCES
POMONA, CA 91766

DU, FEI, PHD
ASSOCIATE PROFESSOR
DEPARTMENT OF PSYCHIATRY
MCLEAN HOSPITAL
HARVARD MEDICAL SCHOOL
BELMONT, MA 02478

DUAN, XIN, PHD
ASSOCIATE PROFESSOR
DEPARTMENT OF OPTHOMOLGY
UNIVERSITY OF CALIFORNIA, SAN FRANCISCO
SCHOOL OF MEDICINE
SAN FRANCISCO, CA 94158

ELLISEN, LEIF W, PHD, MD
PROFESSOR AND PROGRAM DIRECTOR
CENTER FOR BREAST CANCER
MASSACHUSETTS GENERAL HOSPITAL CANCER CENTER
BOSTON, MA 02114

EVANS, TODD R, PHD
PROFESSOR
DEPARTMENT OF SURGERY
ASSOCIATE DEAN FOR RESEARCH
WEILL CORNELL MEDICAL COLLEGE
CORNELL UNIVERSITY
NEW YORK, NY 10065

EZASHI, TOSHIHIKO, PHD
RESEARCH PROFESSOR
COLORADO CENTER FOR REPRODUCTIVE MEDICINE
LONE TREE, CO 80124

FERRARI, GIOVANNI, PHD
ASSOCIATE PROFESSOR
DEPARTMENT OF SURGERY
COLUMBIA BIOBANK FOR TRANSLATIONAL SCIENCE
COLUMBIA UNIVERSITY IRVING MEDICAL CENTER
COLUMBIA UNIVERSITY
NEW YORK, NY 10032

FITZGERALD, MICHAEL C, PHD
PROFESSOR
DEPARTMENT OF CHEMISTRY
DUKE UNIVERSITY
DURHAM, NC 27708

FLORES, ELSA R, PHD
ASSOCIATE CENTER DIRECTOR AND DIVISION CHIEF, BASIC SCIENCE
DEPARTMENT OF MOLECULAR ONCOLOGY
H. LEE MOFFITT CANCER CENTER
AND RESEARCH INSTITUTE
TAMPA, FL 33612

FOSTER, MARK A, PHD
ASSOCIATE PROFESSOR
DEPARTMENT OF ELECTRICAL ENGINEERING
AND COMPUTING
WHITING SCHOOL OF ENGINEERING
JOHNS HOPKINS UNIVERSITY
BALTIMORE, MD 21218

FOX, NATHAN A, PHD
DISTINGUISHED UNIVERSITY PROFESSOR
DEPARTMENT OF HUMAN DEVELOPMENT
UNIVERSITY OF MARYLAND
COLLEGE PARK, MD 20742

GARNER, AMANDA, PHD
PROFESSOR
COLLEGE OF PHARMACY
DEPARTMENT OF MEDICINAL CHEMISTRY
UNIVERSITY OF MICHIGAN
ANN ARBOR, MI 48109

GARZA, LUIS A, MD, PHD
PROFESSOR
DEPARTMENT OF DERMATOLOGY
JOHNS HOPKINS UNIVERSITY
BALTIMORE, MD 21287

GILL, SAAR, PHD, MD
ASSOCIATE PROFESSOR
DEPARTMENT OF MEDICINE
SMILOW CENTER FOR TRANSLATIONAL RESEARCH
PERELMAN SCHOOL OF MEDICINE
UNIVERSITY OF PENNSYLVANIA
PHILADELPHIA, PA 19104

GILLESPIE, AMANDA I, PHD
ASSOCIATE PROFESSOR
DEPARTMENT OF OTOLARYNGOLOGY
EMORY UNIVERSITY SCHOOL OF MEDICINE
ATLANTA, GA 30308

GINTY, ANNIE T, PHD
ASSOCIATE PROFESSOR
DEPARTMENT OF PSYCHOLOGY AND NEUROSCIENCE
BAYLOR UNIVERSITY
WACO, TX 76798

GIORDANO, NICHOLAS A, PHD
ASSISTANT PROFESSOR
NELL HODGSON WOODRUFF SCHOOL OF NURSING
EMORY UNIVERSITY
ATLANTA, GA 30322

GRIENBERGER, CHRISTINE, PHD
ASSISTANT PROFESSOR
DEPARTMENT OF BIOLOGY
BRANDEIS UNIVERSITY
WALTHAM, MA 02453

GUCCIONE, ERNESTO, PHD
PROFESSOR
DEPARTMENT OF ONCOLOGICAL AND
PHARMACOLOGICAL SCIENCES
HESS CENTER FOR SCIENCE AND MEDICINE
ICAHN SCHOOL OF MEDICINE AT MOUNT SINAI
NEW YORK, NY 10029

GUERRIERO, REJEAN M, DO
ASSOCIATE PROFESSOR
DEPARTMENT OF NEUROLOGY
SCHOOL OF MEDICINE
WASHINGTON UNIVERSITY
ST. LOUIS, MO 63110

GUILLEMIN, KAREN J, PHD
PROFESSOR
DEPARTMENT OF BIOLOGY
INSTITUTE OF MOLECULAR BIOLOGY
UNIVERSITY OF OREGON
EUGENE, OR 97403

GUZMAN, RAUL J, MD
PROFESSOR
CHIEF, DIVISION OF VASCULAR SURGERY
DEPARTMENT OF SURGERY
YALE UNIVERSITY
NEW HAVEN, CT 06520

HAMPSON, MICHELLE, PHD
PROFESSOR
DEPARTMENT OF RADIOLOGY & BIOMEDICAL IMAGING
SCHOOL OF MEDICINE
YALE UNIVERSITY
NEW HAVEN, CT 06520

HARTIG, SEAN, PHD
ASSOCIATE PROFESSOR
DEPARTMENT OF MEDICINE
DIVISION OF ENDOCRINOLOGY, DIABETES AND METABOLISM
DEPARTMENT OF MOLECULAR AND CELL BIOLOGY
BAYLOR COLLEGE OF MEDICINE
HOUSTON, TX 77030

HENDERSON, RORY, PHD
ASSISTANT PROFESSOR
DEPARTMENT OF MEDICINE
DUKE UNIVERSITY
DURHAM, NC 27713

HOCHMAN, SHAWN, PHD
PROFESSOR
DEPARTMENT OF CELL BIOLOGY
SCHOOL OF MEDICINE
EMORY UNIVERSITY
ATLANTA, GA 30322

HODGES, MATTHEW R, PHD
PROFESSOR
DEPARTMENT OF PHYSIOLOGY
MEDICAL COLLEGE OF WISCONSIN
MILWAUKEE, WI 53226

HRIBAR, MICHELLE, PHD
ASSOCIATE PROFESSOR
OPHTHALMOLOGY AT CASEY EYE INSTITUTE AND
DEPARTMENT OF MEDICAL INFORMATICS
AND CLINICAL EPIDEMIOLOGY
OREGON HEALTH & SCIENCE UNIVERSITY
PORTLAND, OR 97239

JACOBS, DAVID R JR, PHD
PROFESSOR
DIVISION OF EPIDEMIOLOGY AND COMMUNITY HEALTH
SCHOOL OF PUBLIC HEALTH
UNIVERSITY OF MINNESOTA
MINNEAPOLIS, MN 55454

JEPSEN, KRISTEN L, PHD
DIRECTOR
INSTITUTE FOR GENOMIC MEDICINE
UNIVERSITY OF CALIFORNIA, SAN DIEGO
LA JOLLA, CA 92093

JI, ZHICHENG, PHD
ASSISTANT PROFESSOR
DEPARTMENT OF BIOSTATISTICS AND BIOINFORMATICS
DUKE UNIVERSITY
DURHAM, NC 27705

JIMENO, ANTONIO, MD, PHD
PROFESSOR OF MEDICINE
DIVISION OF MEDICAL ONCOLOGY
SCHOOL OF MEDICINE
UNIVERSITY OF COLORADO, DENVER
AURORA, CO 80045

KADONAGA, JAMES T, PHD
PROFESSOR
DEPARTMENT OF MOLECULAR BIOLOGY
UNIVERSITY OF CALIFORNIA, SAN DIEGO
LA JOLLA, CA 92093

KAMARCK, THOMAS W, PHD
PROFESSOR OF PSYCHOLOGY AND PSYCHIATRY
DEPARTMENT OF PSYCHOLOGY
UNIVERSITY OF PITTSBURGH
PITTSBURGH, PA 15213

KAMPMANN, MARTIN, PHD
ASSISTANT PROFESSOR
DEPARTMENT OF BIOCHEMISTRY AND BIOPHYSICS
INSTITUTE FOR NEURODEGENERATIVE DISEASES
UNIVERSITY OF CALIFORNIA, SAN FRANCISCO
SAN FRANCISCO, CA 94143

KASINSKI, ANDREA L, PHD
ASSOCIATE PROFESSOR
DEPARTMENT OF BIOLOGICAL SCIENCES
PURDUE UNIVERSITY
WEST LAFAYETTE, IN 47907

KERBER, KEVIN A, MD
PROFESSOR
DEPARTMENT OF NEUROLOGY
THE OHIO STATE UNIVERSITY
COLUMBUS, OH 43210

KESWANI, SUNDEEP G, MD
PROFESSOR AND CLAYTON ENDOWED CHAIR
DIVISION OF PEDIATRIC SURGERY
TEXAS CHILDREN'S HOSPITAL
BAYLOR COLLEGE OF MEDICINE
HOUSTON, TX 77030

KIM, BRIAN, MTR, MD
SOL AND CLARA KEST PROFESSOR
THE KIMBERLY AND ERIC J. WALDMAN DEPARTMENT OF
DERMATOLOGY
ICAHN SCHOOL OF MEDICINE AT MOUNT SINAI
NEW YORK, NY 10019

KING, GAVIN M, PHD
PROFESSOR
DEPARTMENT OF PHYSICS AND ASTRONOMY
UNIVERSITY OF MISSOURI-COLUMBIA
COLUMBIA, MO 65211

KLEIN, EILI Y, PHD
ASSOCIATE PROFESSOR
DEPARTMENT OF EPIDEMIOLOGY
JOHNS HOPKINS
BALTIMORE, MD 21209

KLIMENTIDIS, YANN C, PHD
ASSOCIATE PROFESSOR
EPIDEMIOLOGY AND BIOSTATISTICS DEPARTMENT
UNIVERSITY OF ARIZONA
TUCSON 85724

KONG, NAN, PHD
PROFESSOR
WELDON SCHOOL OF BIOMEDICAL ENGINEERING
SCHOOL OF INDUSTRIAL ENGINEERING
PURDUE UNIVERSITY
WEST LAFAYETTE, IN 47907

KRAMER, DANIEL R, MD
ASSISTANT PROFESSOR
DEPARTMENT OF NEUROSURGERY
UNIVERSITY OF COLORADO
AURORA, CO 80045

KUPPERMANN, MIRIAM, PHD
PROFESSOR
DEPARTMENT OF OBSTETRICS, GYNECOLOGY
AND REPRODUCTIVE SCIENCES
UNIVERSITY OF CALIFORNIA
SAN FRANCISCO, CA 94143

LAIDLAW, BRIAN, PHD
ASSISTANT PROFESSOR
DEPARTMENTS OF MEDICINE, PATHOLOGY AND
IMMUNOLOGY
WASHINGTON UNIVERSITY SCHOOL OF MEDICINE
ST. LOUIS, MO 63110

LEI, LEI, PHD
ASSOCIATE PROFESSOR
DEPARTMENT OF OBSTETRICS, GYNECOLOGY AND
WOMEN'S
UNIVERSITY OF MISSOURI SCHOOL OF MEDICINE
COLUMBIA, MO 65211

LESSARD, CHRISTOPHER J, PHD
PROFESSOR
GENES AND HUMAN DISEASE RESEARCH PROGRAM
OKLAHOMA MEDICAL RESEARCH FOUNDATION
OKLAHOMA CITY, OK 73104

LEVINSON, CHERI A, PHD
PROFESSOR
DEPARTMENT OF PSYCHOLOGY
UNIVERSITY OF LOUISVILLE
LOUISVILLE, KY 40292

LI, YONG, PHD
PROFESSOR
DEPARTMENT OF MEDICINE
SECTION OF EPIDEMIOLOGY AND POPULATION SCIENCES
DAN L DUNCAN COMPREHENSIVE CANCER CENTER
BAYLOR COLLEGE OF MEDICINE
HOUSTON, TX 77030

LIANG, BO, PHD
ASSOCIATE PROFESSOR
DEPARTMENT OF BIOCHEMISTRY
EMORY UNIVERSITY SCHOOL OF MEDICINE
ATLANTA, GA 30322

LIAO, ZHONGXING, MD
PROFESSOR
DEPARTMENT OF RADIATION ONCOLOGY
UNIVERSITY OF TEXAS MD ANDERSON CANCER CENTER
HOUSTON, TX 77030

LIM, MEGAN S, MD, PHD
ATTENDING PATHOLOGIST
DEPARTMENT OF PATHOLOGY AND LABORATORY
MEDICINE
LYMPHOMA TRANSLATIONAL RESEARCH PROGRAM
MEMORIAL SLOAN KETTERING CANCER CENTER
NEW YORK, NY 10065

LIU, JIANDONG, PHD
PROFESSOR
DEPARTMENT OF PATHOLOGY AND LABORATORY
MEDICINE
MCALLISTER HEART INSTITUTE
UNIVERSITY OF NORTH CAROLINA AT CHAPEL HILL
CHAPEL HILL, NC 27599

LOSIN, ELIZABETH R, PHD
DEPARTMENT OF CARING AND COMPASSION IN
ADULTHOOD
PENNSYLVANIA STATE UNIVERSITY
CORAL GABLES, FL 33146

LUMENG, CAREY N, MD, PHD
PROFESSOR
DEPARTMENT OF PEDIATRICS
UNIVERSITY OF MICHIGAN MEDICAL SCHOOL
ANN ARBOR, MI 48109

LYUBCHENKO, YURI L, PHD, DSC
PROFESSOR
DEPARTMENT OF PHARMACEUTICAL SCIENCES
DIRECTOR, NANOIMAGING CORE FACILITY
COLLEGE OF PHARMACY
UNIVERSITY OF NEBRASKA MEDICAL CENTER
OMAHA, NE 68198

MAGNOTTA, VINCENT A, PHD
PROFESSOR
DEPARTMENT OF RADIOLOGY
DIVISION OF NEURORADIOLOGY
CARVER COLLEGE OF MEDICINE
UNIVERSITY OF IOWA
IOWA CITY, IA 52242

MALANDRAKI, GEORGIA, PHD
PROFESSOR
DEPARTMENT OF SPEECH, LANGUAGE AND
HEARING SCIENCES
PURDUE UNIVERSITY
WEST LAFAYETTE, IN 47907

MANGALAM, ASHUTOSH K, PHD
ASSOCIATE PROFESSOR
DEPARTMENT OF PATHOLOGY
UNIVERSITY OF IOWA
IOWA CITY, IA 52242

MAURO, THEODORA M, MD
PROFESSOR
DEPARTMENT OF DERMATOLOGY
UNIVERSITY OF CALIFORNIA, SAN FRANCISCO
SAN FRANCISCO, CA 94121

MAX, JEFFREY E, MBBCH
PROFESSOR
UNIVERSITY OF CALIFORNIA, SAN DIEGO
SAN DIEGO, CA 92121

MEDINA, LUIS D, PHD
ASSOCIATE PROFESSOR
DEPARTMENT OF PSYCHOLOGY
UNIVERSITY OF HOUSTON
HOUSTON, TX 77204

MILLER, JORDAN D, PHD
ASSOCIATE PROFESSOR
DEPARTMENT OF CARDIOVASCULAR SURGERY
MAYO CLINIC
ROCHESTER, MN 55905

MILLS, ALEA A, PHD
PROFESSOR
DEPARTMENT OF MOLECULAR BIOLOGY AND GENETICS
COLD SPRING HARBOR LABORATORY
COLD SPRING HARBOR, NY 11724

MITTAG, TANJA, PHD
MEMBER
DEPARTMENT OF STRUCTURAL BIOLOGY
ST. JUDE CHILDREN'S RESEARCH HOSPITAL
MEMPHIS, TN 38105

MOSER, EMILY K, PHD
ASSISTANT PROFESSOR
DEPARTMENT OF MEDICINE
DIVISION OF PULMONARY, CRITICAL CARE AND SLEEP
GAINESVILLE, FL 32610

MURRAY, STUART B, PHD
ASSOCIATE PROFESSOR
DEPARTMENT OF PSYCHIATRY AND BEHAVIORAL
SCIENCES
KECK SCHOOL OF MEDICINE
UNIVERSITY OF SOUTHERN CALIFORNIA
LOS ANGELES, CA 90033

MYUNG, PEGGY S, PHD, MD
ASSOCIATE PROFESSOR OF DERMATOLOGY AND
PATHOLOGY
YALE UNIVERSITY
NEW HAVEN, CT 06511

NAKANISHI, KOTARO, SCD
PROFESSOR
THE OHIO STATE UNIVERSITY
COLUMBUS, OH 43210

NEUNER, JOAN M, MD
PROFESSOR
DEPARTMENT OF GENERAL INTERNAL MEDICINE
CENTER FOR PATIENT CARE AND OUTCOMES RESEARCH
MEDICAL COLLEGE OF WISCONSIN
MILWAUKEE, WI 53226

NEVILLE, THANH H, MD
ASSOCIATE PROFESSOR
DIVISION OF PULMONARY & CRITICAL CARE
DAVID GEFFEN SCHOOL OF MEDICINE AT UCLA
LOS ANGELES, CA 90095

OCASIO, MANUEL A, PHD
ASSISTANT PROFESSOR
DEPARTMENT OF SOCIAL, BEHAVIORAL, AND
POPULATION SCIENCES
SCHOOL OF PUBLIC HEALTH AND TROPICAL MEDICINE
TULANE UNIVERSITY
NEW ORLEANS, LA 70112

OKUN, MICHAEL S, FELLOWSHIP, MD
PROFESSOR
DEPARTMENT OF NEUROLOGY
UNIVERSITY OF FLORIDA
GAINESVILLE, FL 32610

OUYANG, YINGSHI, PHD
ASSISTANT PROFESSOR
MAGEE-WOMEN’S RESEARCH INSTITUTE
UNIVERSITY OF PITTSBURGH SCHOOL OF MEDICINE
PITTSBURGH, PA 15213

PAN, JIANJUN, PHD
ASSOCIATE PROFESSOR
DEPARTMENT OF PHYSICS
UNIVERSITY OF SOUTH FLORIDA
TAMPA, FL 33647

PAPAPETROU, EIRINI, MD, PHD
ASSOCIATE PROFESSOR
DEPARTMENT OF MEDICINE
DIVISION OF HEMATOLOGY AND ONCOLOGY
ICAHN SCHOOL OF MEDICINE AT MOUNT SINAI
NEW YORK, NY 10029

PARAST, MANA M, MD, PHD
PROFESSOR
DEPARTMENT OF PATHOLOGY
SCHOOL OF MEDICINE
SANFORD CONSORTIUM FOR REGENERATIVE MEDICINE
UNIVERSITY OF CALIFORNIA-SAN DIEGO
LA JOLLA, CA 92093

PARROTT, DOMINIC, PHD
PROFESSOR
DEPARTMENT OF PSYCHOLOGY
GEORGIA STATE UNIVERSITY
ATLANTA, GA 30302

PATEL, MANOJ K, PHD
ASSOCIATE PROFESSOR
DEPARTMENT OF ANESTHESIOLOGY
UNIVERSITY OF VIRGINIA HEALTH SYSTEM
CHARLOTTESVILLE, VA 22908

PAULOS, CHRYSTAL M, PHD
DAVID H. LAWSON, MD PROFESSOR OF CANCER
RESEARCH ASSOCIATE PROFESSOR OF SURGERY
DIRECTOR TRANSLATIONAL RESEARCH
WINSHIP CANCER INSTITUTE OF EMORY UNIVERSITY
EMORY UNIVERSITY SCHOOL OF MEDICINE
ATLANTA, GA 30322

PIERRE, JOSEPH F, PHD
ASSISTANT PROFESSOR
DEPARTMENT OF NUTRITIONAL SCIENCES
UNIVERSITY OF WISCONSIN – MADISON
MADISON, WI 53706

PINE, SHARON R, PHD
ASSOCIATE PROFESSOR
DEPARTMENT OF MEDICINE
DIVISION OF MEDICAL ONCOLOGY
UNIVERSITY OF COLORADO SCHOOL OF MEDICINE
AURORA, CO 80045

PITTS, TERESA G, PHD
ASSOCIATE PROFESSOR
SPEECH, LANGUAGE, HEARING SCIENCES
UNIVERSITY OF MISSOURI
COLUMBIA, MO 65211

PLAXCO, KEVIN W, PHD
PROFESSOR
DEPARTMENT OF CHEMISTRY AND BIOCHEMISTRY
UNIVERSITY OF CALIFORNIA, SANTA BARBARA
SANTA BARBARA, CA 93106

PLOWMAN, EMILY K, PHD
PROFESSOR
DEPARTMENT OF OTOLARYNGOLOGY-HEAD AND NECK SURGERY
DIRECTOR, AERODIGESTIVE RESEARCH CORE &
WEXNER MEDICAL CENTER DYSPHAGIA PROGRAM
THE OHIO STATE UNIVERSITY
COLUMBUS, OH 43212

POLLACK, ANNA Z, PHD
ASSOCIATE PROFESSOR
DEPARTMENT OF GLOBAL AND
COMMUNITY HEALTH
GEORGE MASON UNIVERSITY
FAIRFAX, VA 22030

POLLACK, LOIS, PHD
PROFESSOR
SCHOOL OF APPLIED
AND ENGINEERING PHYSICS
CORNELL UNIVERSITY
ITHACA, NY 14853

QI, LEI STANLEY, PHD
ASSOCIATE PROFESSOR
DEPARTMENTS OF BIOENGINEERING, AND
SARAFAN CHEM-H
STANFORD UNIVERSITY
STANFORD, CA 94305

RAGHAVEN, MALINI, PHD
PROFESSOR
DEPARTMENT OF MICROBIOLOGY AND IMMUNOLOGY
UNIVERSITY OF MICHIGAN MEDICAL SCHOOL
ANN ARBOR, MI 48109

RAISON, CHARLES, MD
PROFESSOR
DEPARTMENT OF PSYCHIATRY
SCHOOL OF MEDICINE AND PUBLIC HEALTH
UNIVERSITY OF WISCONSIN-MADISON
MADISON, WI 53713

RAPER, JESSICA, PHD
ASSOCIATE PROFESSOR
DEVELOPMENTAL AND COGNITIVE NEUROSCIENCE
YERKES NATIONAL PRIMATE RESEARCH CENTER
EMORY UNIVERSITY
ATLANTA, GA 30322

RECHT, LAWRENCE D, MD
PROFESSOR
DEPARTMENT OF NEUROLOGY
STANFORD UNIVERSITY MEDICAL CENTER
STANFORD, CA 94305

RENTHAL, WILLIAM R, MD, PHD
ASSOCIATE PROFESSOR OF NEUROLOGY
DEPARTMENT OF NEUROLOGY
BRIGHAM AND WOMEN'S HOSPITAL
HARVARD MEDICAL SCHOOL
BOSTON, MA 02115

REYNOLDS, JOSEPH M, PHD
ASSOCIATE PROFESSOR AND CHAIR OF MICROBIOLOGY AND IMMUNOLOGY
DEPARTMENT OF MICROBIOLOGY AND IMMUNOLOGY
ROSALIND FRANKLIN UNIV OF MEDICINE & SCI
NORTH CHICAGO, IL 60064

RIBAS, ANTONI, MD, PHD
PROFESSOR OF MEDICINE AND SURGERY
DIVISION OF HEMATOLOGY AND ONCOLOGY
JOHNSON COMPREHENSIVE CANCER CENTER
UNIVERSITY OF CALIFORNIA LOS ANGELES
LOS ANGELES, CA 90095

RISKA, KRISTAL M, PHD
ASSISTANT PROFESSOR
DEPARTMENT OF SURGERY
DIVISION OF HEAD AND NECK SURGERY
AND COMMUNICATION SCIENCES
DUKE UNIVERSITY SCHOOL OF MEDICINE
DURHAM, NC 27710

ROBSON, PAUL, PHD
PROFESSOR
THE JACKSON LABORATORY FOR GENOMIC MEDICINE
FARMINGTON, CT 06032

ROMBERG, NEIL D, MD
ASSOCIATE PROFESSOR
DEPARTMENT OF IMMUNOLOGY SERVICE
CHILDREN'S HOSPITAL OF PHILADELPHIA
UNIVERSITY OF PENNSYLVANIA
PHILADELPHIA, PA 19104

RYAN, ALICE S, PHD
PROFESSOR OF MEDICINE
DIVISION OF GERONTOLOGY, GERIATRICS, AND PALLIATIVE MEDICINE
SCHOOL OF MEDICINE
UNIVERSITY OF MARYLAND
BALTIMORE, MD 21201

SADEGHI GHANDEHARI, SOROUSH, PHD, MD
ASSISTANT PROFESSOR
DEPARTMENT OF OTOLARYNGOLOGY -
HEAD AND NECK SURGERY
JOHNS HOPKINS UNIVERSITY
JOHNS HOPKINS UNIVERSITY
BALTIMORE, MD 21205

SAGGAR, MANISH, PHD
ASSISTANT PROFESSOR
DEPARTMENT OF PSYCHIATRY
SCHOOL OF MEDICINE
STANFORD UNIVERSITY
PALO ALTO, CA 94304

SCHUST, DANNY J, MD
PROFESSOR
VICE CHAIR IN THE DEPARTMENT OF OBSTETRICS AND GYNECOLOGY
DUKE UNIVERSITY
DURHAM, NC 27708

SHECHTER, ARI, PHD
ASSOCIATE PROFESSOR
DEPARTMENT OF MEDICINE
CENTER FOR BEHAVIORAL CARDIOVASCULAR HEALTH
COLUMBIA UNIVERSITY MEDICAL CENTER
NEW YORK, NY 10128

SHI, TUJIN, PHD
SENIOR RESEARCH SCIENTIST
DIVISION OF BIOLOGICAL SCIENCES
PACIFIC NORTHWEST NATIONAL LABORATORY
RICHLAND, WA 99352

SILBERSTEIN, LEV, MD, PHD
ASSOCIATE PROFESSOR
TRANSLATIONAL SCIENCE AND THERAPEUTICS DIVISION
FRED HUTCHINSON CANCER CENTER
SEATTLE, WA 98109

SIMPSON, ELIZABETH A, PHD
ASSOCIATE PROFESSOR
ASSISTANT PROFESSOR
DEPARTMENT OF PSYCHOLOGY, CHILD DIVISION
UNIVERSITY OF MIAMI CORAL GABLES
CORAL GABLES, FL 33124

SOLDNER, FRANK, MD
ASSISTANT PROFESSOR
DEPARTMENT OF NEUROSCIENCE AND GENETICS
ALBERT EINSTEIN COLLEGE OF MEDICINE
BRONX, NY 10461-1975

SONKUSALE, SAMEER R, PHD
PROFESSOR
DEPARTMENT OF ELECTRICAL AND
COMPUTER ENGINEERING
TUFTS UNIVERSITY
MEDFORD, MA 02155

SWEEDLER, JONATHAN V, PHD
PROFESSOR
DEPARTMENT OF CHEMISTRY
UNIVERSITY OF ILLINOIS, URBANA-CHAMPAIGN
URBANA, IL 61801

TEIJARO, JOHN R, PHD
PROFESSOR
DEPARTMENT OF IMMUNOLOGY AND MICROBIAL SCIENCE
THE SCRIPPS RESEARCH INSTITUTE
LA JOLLA, CA 92122

THOMAS, JENNIFER J, PHD
ASSOCIATE PROFESSOR
DEPARTMENT OF PSYCHIATRY
MASSACHUSETTS GENERAL HOSPITAL
HARVARD MEDICAL SCHOOL
BOSTON, MA 02114

TOM, VERONICA J, PHD
PROFESSOR
DEPARTMENT OF NEUROBIOLOGY AND
ANATOMY
DREXEL UNIVERSITY COLLEGE OF MEDICINE
PHILADELPHIA, PA 19129

TREBLE-BARNA, AMERY, PHD
ASSISTANT PROFESSOR
DEPARTMENT OF PHYSICAL MEDICINE & REHABILITATION
SCHOOL OF MEDICINE
UNIVERSITY OF PITTSBURGH
PITTSBURGH, PA 15224

TSAO, BETTY P, PHD
PROFESSOR
DIVISION OF RHEUMATOLOGY AND IMMUNOLOGY
DEPARTMENT OF MEDICINE
MEDICAL UNIVERSITY OF SOUTH CAROLINA
CHARLESTON, SC 29425

TURNER, ROBERT S, PHD
PROFESSOR
DEPARTMENT OF NEUROBIOLOGY
SYSTEMS NEUROSCIENCE CENTER
UNIVERSITY OF PITTSBURGH
PITTSBURGH, PA 15261

UDDIN, MONICA, PHD
PROFESSOR
GENOMICS PROGRAM
COLLEGE OF PUBLIC HEALTH
UNIVERSITY OF SOUTH FLORIDA
TAMPA, FL 33612

VAUGHAN, ANDREW, PHD
ASSOCIATE PROFESSOR
DEPARTMENT OF BIOMEDICAL SCIENCES
INSTITUTE FOR REGENERATIVE MEDICINE
SCHOOL OF VETERINARY MEDICINE
UNIVERSITY OF PENNSYLVANIA
PHILADELPHIA, PA 19104

VERDOLINI, KATHERINE, PHD
PROFESSOR
DEPARTMENT OF COMMUNICATION SCIENCES
AND DISORDERS
UNIVERSITY OF DELAWARE
NEWARK, DE 19713

WANG, CHANGLU, PHD
EXTENSION SPECIALIST
DEPARTMENT OF ENTOMOLOGY
RUTGERS UNIVERSITY
EAST BRUNSWICK, NJ 08815

WANG, JING, PHD
PROFESSOR
DEPARTMENT OF RADIATION ONCOLOGY
UNIVERSITY OF TEXAS SOUTHWESTERN MEDICAL CENTER
DALLAS, TX 75235

WANG, JING, PHD
PROFESSOR
DEPARTMENT OF CANCER BIOLOGY AND GENETICS
THE OHIO STATE UNIVERSITY WEXNER MEDICAL CENTER
COLUMBUS, OH 43210

WESSEL, GARY M, PHD
PROFESSOR
DEPARTMENT OF MOLECULAR BIOLOGY CELL BIOLOGY
AND BIOCHEMISTRY
BROWN UNIVERSITY
PROVIDENCE, RI 02912

WHITEHEAD, TIMOTHY A, PHD
PROFESSOR
DEPARTMENT OF CHEMICAL
AND BIOLOGICAL ENGINEERING
UNIVERSITY OF COLORADO BOULDER
BOULDER, CO 80309

WILDES, JENNIFER E, PHD
PROFESSOR
DEPARTMENT OF PSYCHIATRY AND
BEHAVIORAL NEUROSCIENCE
UNIVERSITY OF CHICAGO
CHICAGO, IL 60637

WOLF, ERIKA J, PHD
PROFESSOR
DEPARTMENT OF PSYCHIATRY
SCHOOL OF MEDICINE
BOSTON UNIVERSITY
BOSTON 02118

YESSELMAN, JOSEPH, PHD
ASSISTANT PROFESSOR
DEPARTMENT OF CHEMISTRY
UNIVERSITY OF NEBRASKA-LINCOLN
LINCOLN, NE 68588

YOUNG, KYM, PHD
ASSOCIATE PROFESSOR
DEPARTMENT OF PSYCHIATRY
UNIVERSITY OF PITTSBURGH
PITTSBURGH, PA 15213

ZENG, JING, MD
ASSOCIATE PROFESSOR
DEPARTMENT OF RADIATION ONCOLOGY
UNIVERSITY OF WASHINGTON, SEATTLE
SEATTLE, WA 98195

ZHAO, YONGXIN, PHD
EBERLY FAMILY ASSOCIATE PROFESSOR OF BIOLOGICAL SCIENCES
DEPARTMENT OF BIOLOGICAL SCIENCES
CARNEGIE-MELLON UNIVERSITY
PITTSBURGH, PA 15213

ZHOU, HUAN-XIANG, PHD
PROFESSOR
DEPARTMENTS OF CHEMISTRY AND PHYSICS
UNIVERSITY OF ILLINOIS AT CHICAGO
CHICAGO, IL 60607

SCIENTIFIC REVIEW OFFICER

MASON-MAH, BRITTANY L, PHD
SCIENTIFIC REVIEW OFFICER
CENTER FOR SCIENTIFIC REVIEW
NATIONAL INSTITUTES OF HEALTH
BETHESDA, MD 20892

EXTRAMURAL SUPPORT ASSISTANT

GRUCELLA, BRANDY L
EXTRAMURAL SUPPORT ASSISTANT
CENTER FOR SCIENTIFIC REVIEW
NATIONAL INSTITUTES OF HEALTH
BETHESDA, MD 20892

HILL, DANA K
EXTRAMURAL SUPPORT ASSISTANT
CENTER FOR SCIENTIFIC REVIEW
NATIONAL INSTITUTES OF HEALTH
BETHESDA, MD 20892

Consultants are required to absent themselves from the room during the review of any application if their presence would constitute or appear to constitute a conflict of interest.
