# Supplementary material for: Rapid Innovation of Precision Psychiatry Interventions Using Dynamical Systems Modeling and Ecological Quasi-Experiments: Protocol for a Proof-of-Concept Study on Eating Disorders
Source: JMIR Res Protoc. 2026 Oct 15;15:e104849. doi: 10.2196/104849 (PMC13671667; doi:10.2196/104849)
Supplement: Supplementary file 2 [file resprot-v15-e104849-s001.docx]

*Table 1. Sample just-in-time adaptive intervention (JITAI) content*

| **CBT-E Skill** | **Treatment Strategy** | **Example JITAI Text** |
| --- | --- | --- |
| Reducing Dietary Restraint | Eating regular meals and snacks | **EDDI is detecting possible increased risk for an eating disorder behavior.**  Dietary restraint can interfere with quality of life and impact your mood, health, and relationships. Have you eaten every 3-4 hours so far today? If not, remember why it matters to you to reduce dietary restriction.  *Action Step:* If it has been more than 3 hours, eat your next meal or snack soon. Make and follow a plan to eat every 3-4 hours for the rest of the day. Click here to view your regular eating schedule and a quick summary! |
| Urge Management | Urge surfing | **EDDI is detecting possible increased risk for an eating disorder behavior.**  Remember that urges to restrict, binge eat, or use unhealthy weight control behaviors will come and go. Although it can feel like you must automatically respond to these urges to get them to go away, urges will eventually go away on their own with time.  *Action Step:* Reflect on any urges you are currently experiencing and rate their intensity from 0-10. Set a timer for 15 minutes and use urge surfing to ride out the urge. When your timer goes off, rate the urge intensity again. Repeat if needed until the urge is manageable. Click here to view a quick summary of common urges and how to urge surf. |
| Emotion Regulation | Alternative Activities | **EDDI is detecting possible increased risk for an eating disorder behavior.**  Enjoyable alternative activities can significantly boost your mood. This means that alternative activities can help you navigate negative emotions without engaging in eating disorder behaviors.  *Action Step:* Pick out an enjoyable alternative activity that you can use now. Engage in the activity for the next 15-20 minutes. Click here to view a list of potential alternative activities. |
| Improving Body Image | Reducing body checking | **EDDI is detecting possible increased risk for an eating disorder behavior.**  Shape checking, like scrutinizing yourself in the mirror, measuring or pinching yourself, or noticing how clothes fit, keeps you focused on small, short-lived changes in your shape. These behaviors can reinforce body image concerns and eating disorder behaviors.  *Action Step:* Identify body checking behaviors that you have engaged in today. Make a commitment to notice urges for and avoid those behaviors for the rest of the day. Click here to view a list of checking behaviors. |

Note: All JITAIs will include hyperlinks to the in-app skills repository and reflection activities participants complete while watching the video modules.
